# Evolution of biological cooperation: an algorithmic approach

**DOI:** 10.1038/s41598-024-52028-0

**Published:** 2024-01-17

**Authors:** Ivan Sudakow, John Reinitz, Sergey A. Vakulenko, Dima Grigoriev

**Affiliations:** 1 School of Mathematics and Statistics, The Open University, Milton Keynes, MK7 6AA UK; 2 Departments of Statistics, Ecology and Evolution, Molecular Genetics and Cell Biology, University of Chicago, Chicago, 10587 IL USA; 3 Institute for Problems in Mechanical Engineering, Russian Academy of Sciences, Saint Petersburg, 199178 Russia; 4 Saint Petersburg Electrotechnical University, Saint Petersburg, 197022 Russia; 5 CNRS, Mathématiques, Université de Lille, Villeneuve d’Ascq, Lille, 59655 France

**Keywords:** Applied mathematics, Evolutionary theory

## Abstract

This manuscript presents an algorithmic approach to cooperation in biological systems, drawing on fundamental ideas from statistical mechanics and probability theory. Fisher’s geometric model of adaptation suggests that the evolution of organisms well adapted to multiple constraints comes at a significant complexity cost. By utilizing combinatorial models of fitness, we demonstrate that the probability of adapting to all constraints decreases exponentially with the number of constraints, thereby generalizing Fisher’s result. Our main focus is understanding how cooperation can overcome this adaptivity barrier. Through these combinatorial models, we demonstrate that when an organism needs to adapt to a multitude of environmental variables, division of labor emerges as the only viable evolutionary strategy.

## Introduction

The explanation of organismal development, starting from simple compounds and progressing to complex multicellular forms, presents a significant challenge for the fields of mathematics and physics^1–3^. In this manuscript, we employ an algorithmic approach to elucidate the emergence of cooperation within cell communities. Our proposal entails a mathematical model that demonstrates how the emergence of new cooperation can be effectively explained using Hard Combinatorial models. These models and our approach arise naturally when we assume that gene regulation is governed by networks resembling neural nets with Boolean inputs-a widely accepted assumption supported by notable works (e.g.,^4–7^). Consequently, the optimization of fitness becomes a hard combinatorial problem.

Such problems are of great importance in various applications and have been extensively studied^8–10^. It is widely recognized that many of these problems are inherently difficult, often classified as NP-hard^8^. Due to the impracticality of exhaustive search in such cases, specialized or approximation algorithms must be employed. Notably, Sewall Wright was the first to realize the immense challenge of finding the optimal fitness value within a multidimensional hypercube (genotype space)^11^. Recent publications, such as^12^, delve into the consideration of epistasis effects. In^12^, it is observed that the mutation graph defines the proximity between genotypes, where a genotype *A* is considered close to *B* if *B* can be transformed into *A* through a single mutation. The presence of epistasis and gene pleiotropy (gene incompatibility) renders the search for fitness landscape peaks a hard combinatorial problem^5,6,12,13^.

Our approach can be outlined as follows. We utilize genotype-fitness maps, also known as the fitness landscape ($s \rightarrow F(s)$). These maps play a crucial role in the mathematical description of evolution using an algorithmic approach. However, the structure of these maps remained unknown until the last few decades. Nowadays, real fitness landscapes are being analyzed by constructing genotypes with all possible combinations of small sets of mutations observed in evolution experiments^14–17^. We employ models that are consistent with this data.

Furthermore, it is worth noting that the human genome comprises approximately 22,000 protein-coding genes, a number that is comparable to the genomes of fruit flies and nematodes. Surprisingly, more complex organisms do not necessitate a higher number of genes, despite having a greater number of phenotypic traits and the need to adapt to numerous environmental constraints. These facts challenge the classical ideas of modern evolutionary synthesis. According to the celebrated Fisher geometric model, it can be demonstrated that the likelihood of improving fitness through random mutations diminishes as the organism’s complexity increases^18^. Building upon Fisher’s approach, Orr estimated the adaptation rate $R_e = \frac{d\log F}{dt}$ as a function of the number of environmental constraints *M*, with *F* representing the average population fitness^19^. Orr’s findings indicate that the adaptation rate becomes exponentially small as $M \gg 1$. We refer to this effect as the ’adaptivity (complexity) barrier.’ Thus, for large values of *M*, the adaptivity barrier increases exponentially. Defining the complexity of a real organism or an adaptation in rigorous mathematical terms is challenging. However, in our formal approach, the adaptation complexity is simply represented by the number *M*.

Using various genotype-fitness map models, including one from^20^ that generalizes the Fisher model and classical methods^21–23^, we demonstrate that they all exhibit the same effect: the complexity cost exponentially increases with *M*. This complexity cost effect arises due to pleiotropy, which becomes inevitable when $M \gg N$, where *N* represents the number of genes. When there are more traits to be regulated by the same set of genes, higher pleiotropy occurs, which refers to the average number of unrelated traits affected by a single gene. Pleiotropy is a fundamental characteristic of gene regulation^24^ and one of the primary causes of the complexity cost^19^. The substantial complexity cost results from the pronounced roughness of the fitness landscape, which exhibits numerous peaks, valleys, and ridges connecting those peaks. Notably, fundamental findings on hard combinatorial problems (refer to^10^ for an overview) demonstrate that combinatorial models effectively depict real fitness landscapes, characterized by the presence of multiple peaks, valleys, and connecting ridges^14–17^. It is important to note that studies of fitness landscapes in natural populations have revealed the prevalence of low fitness for intermediate phenotypes, indicating the existence of valleys in the fitness landscape^14–17^. The models considered in this manuscript are capable of describing such landscapes.

First, we consider homeostasis as a set of coordinated biochemical processes that maintain the parameters of an organism within a given domain (see the “Supplementary Material (SM)” and^25^ for details). Next, we derive a relation for the probability of the parameters being within that viability domain and find that this probability’s dependence on the genotype is described by the fitness function proposed in^20^. Maximizing this function leads to challenging combinatorial problems, for which we identify adaptivity barriers. However, we demonstrate that these barriers can be overcome through cooperation. Our combinatorial models demonstrate that the division of labor becomes the only viable evolutionary strategy when an organism must adapt to a multitude of environmental variables. We also investigate the adaptivity barriers associated with the following in *silico models*:

**A**: Simplest unicellular organisms (prokaryotes);
**B**: Cooperation among simple organisms, such as symbiosis of prokaryotes;
**C**: Bacterial colonies with a simple genetic regulation based on greedy principle;
**D**: Cell colonies with a more sophisticated genetic regulation, representing primitive analogs of multicellular organisms.

An example of extreme cooperation is observed in methane-producing colonies, as discussed in^26,27^. The process of decomposing organic matter into methane and carbon dioxide is widespread. According to^26^, the limited availability of energy in methanogenic conversion necessitates efficient cooperation among microorganisms. This mutual dependence, driven by energy constraints, reaches a level where neither partner can function independently. However, when working in unison, they sustain the metabolic activity necessary for survival.

The primary consequence of these mathematical findings is that the transition to cooperation becomes essential for survival when an environment rapidly becomes more challenging. In such cases, cooperative organisms have a higher chance of survival. This assertion can be exemplified using a simple example. Let’s consider a colony of non-interacting cells with 500 genes, which must fulfill 500 ecological constraints, and let’s assume the adaptivity barrier $\alpha =M/N$ is set at $\alpha =1$. Now, suppose an ecological catastrophe occurs, resulting in the emergence of 100 new additional constraints (which form a constraint set, disjoint with previous constraints). In order to adapt, evolution would need to generate approximately 100 new genes, which is highly improbable within a short evolutionary timeframe. However, if we consider a group of cooperating cells, where each cell can handle 100 constraints, only six distinct cell types would be sufficient to adapt to all the constraints, including both the new and pre-existing ones. Note that in many cases $\alpha$ determines the probability of adaptation. This parameter has the order 1 as $M, N>>1$. Basic results (see^10^, Ch. 14) on the existence of phase transitions in hard combinatorial problems show that there are even more remarkable effects in cooperation. Suppose, for example, that to be adapted, an organism must satisfy, say, 1000 constraints with 950 genes, and the adaptivity barrier is $\alpha = 1$. Then the chances of survival are vanishingly small because $950 < 1000$, and these chances decrease exponentially with $M - \alpha N$. However, if due to cooperation the number of restrictions is reduced even slightly, for example to 930, the chances of survival become close to 1. Thus, it may occur that even weak cooperation is capable of saving a population in harsh conditions. Certainly, if the organism fails to find suitable neighbors, it may die.

In conclusion, it is worth noting that there are other approaches that explain the origins of multicellularity and cooperation through various factors, including defense against infectious agents and interactions between hosts and parasites^28,29^, see also^30^ for a review of of different hypotheses and their biological testing.

## Results

The homeostasis problem for a system of chemical kinetics, which can describe metabolic regulation in bacteria (see Sect. Methods), is investigated. We aim to estimate the probability of viability, denoted as $P_{v, T}$, which represents the likelihood that the system parameters remain within a homeostasis domain over a large time period [0, *T*]. To estimate $P_{v, T}$, we employ a novel concept of replicative stability through genetic regulation, as proposed in^25^. Briefly, the concept is as follows: In the work referenced as^31^, it is hypothesized that all cells with fixed genomes and genetic regulations will eventually perish due to external and internal fluctuations. However, if these cells replicate and modify their genome, then the resulting chain of replicating cells can potentially survive indefinitely with a non-zero probability. This hypothesis was mathematically analyzed in^25^, and we have incorporated these results into our study.

By considering all possible situations of stress impact on a bacterium or a cell, we obtain an integral representation for $P_{v, T}$.

This representation exhibits intriguing properties. The chances of viability depend singularly on the efficiency of the gene regulation network (GRN), the rate of innovation within this GRN, and the size of the GRN. The size of a gene regulation network (GRN) is defined by the number $O(N_{reg})$ of nodes it contains. Given that the connectivity of real networks is bounded, the number of network interconnections has the order $O(N_{reg})$. The efficiency of a GRN is characterized by models described in the SM and in^25^. We hypothesize that the GRN network counteracts stress-induced perturbations through appropriate gene expression, with network efficiency quantitatively measuring such compensation (details can be found in the SM).

The innovation rate refers to the probability that the antistress GRN network will enhance its efficiency during the replication stage. This rate determines the GRN size increase and is substantially lower than the mutation rate because an innovation may result from several mutations.

It is demonstrated that the viability probability, regarded as the fitness function *F*(*s*), relies on the genotype $s = (s_1, s_2, ..., s_N)$, where $s_i \in \{0,1\}$. Here the value $s_i=1$ indicates the expression of a gene.

For sufficiently large $T$, the viability probability can be approximated by the following relations:

2.1 $$\begin{aligned} \log P_{v,T} \approx -\sum _{t=1}^{N_{rep}} P_{out} (s(t), {\textbf{w}}(t)) \end{aligned}$$

where $s(t)$ represents the genotype at the $t$-th replication step, $N_{rep}$ is the number of replication steps within the time interval $[0, T]$, and $P_{out} (s, {\textbf{w}})$ is the probability of exiting the homeostasis domain between subsequent replication steps (which is assumed to be small). The variable ${\textbf{w}}$ refers to gene network parameters, determining various coefficients within the gene regulation network (GRN), such as interaction matrix entries, kinetic rates, thresholds, etc. One has

$$\begin{aligned} P_{out}(s,{\textbf{w}})=1- F(s,{\textbf{w}}), \end{aligned}$$

where $F$ can be interpreted as a fitness function:

2.2 $$\begin{aligned} F(s,{\textbf{w}})= \sum _{k=1}^M b_k f_k(s, {\textbf{w}}). \end{aligned}$$

**Figure 1:**
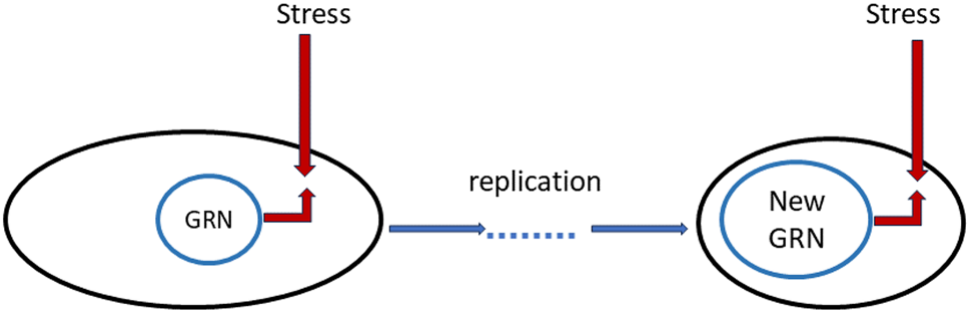
In contrast to physical systems, the stability of biosystems relies on replicative and regulatory mechanisms driven by intricate genetic regulatory networks (GRNs). In the figure, an organism responds to stress using its GRN. There is a ’stress’ arrow directed towards the cell, countered by an arrow from the GRN, which represents the cell’s resistance to the stress. As replication occurs, the GRN gradually expands, resulting in enhanced stress resistance. Leveraging insights from Deep Network theory, we derive an approximate formula to estimate the survival probability of a replicating chain of organisms based on the stress properties, network size, and mutation rate.

Here, $f_k$ represent Boolean functions, and $b_k$ are positive weights. In this context, $M$ represents the number of environmental constraints, corresponding to different stress scenarios, and $f_k$ are probabilities to survive between replications under the $k$-th stress scenario with genotype $s$ and GRN parameters ${\textbf{w}}$. In simpler cases, such as a chain of reactions of the Michaelis–Menten type, this fitness can be reduced to the gene-trait maps, as recently investigated in^20,32,33^. In classical theory (^18,19,34^), the fitness $F$ is fixed, and only the genotype evolves. However, our new idea is as follows: we propose that with replication, the gene regulation network (GRN) expands and enhances stress resistance (see a conceptual scheme in Fig. 1). Determining precise estimates of this resistance is a formidable mathematical problem (see the SM). Nevertheless, we can use estimates from Deep Learning theory, assuming that the efficiency of the GRN depends mainly on its size. Evolution in $t$ involves not only modification of the genotype but also an increase in GRN size step by step, which alters the fitness. This extension of the classical approach explains why evolution is successful and why the GRN expands. In fact, for $f_k$ independent of $t$, we have $P_{v, T} \rightarrow 0$ as $T \rightarrow \infty$, but in our model, evolution can continue indefinitely (see the SM). In fact, for large $T$, we obtain $P_{v, T} \approx 1 - \exp (- c_0 N_{reg}^{\gamma })$, where $P_{v, +\infty }$ represents the probability that the system state remains within a small homeostasis domain over the time interval $(0, +\infty )$. Here, $N_{reg}$ denotes the size of the gene network (i.e., the number of units within the network), $\gamma > 0$ is a small constant influenced by the number of fluctuating parameters of random stress, and $c_0$ is a positive constant depending on details of the system’s chemical kinetics. GRNs compensate for stress impacts (see also^25^). We show that this compensation leads to an exponential increase in the viability probability. The larger the network, the better the compensation, as follows from the above estimate for $P_{v, +\infty }$.

One can show that larger GRNs are better equipped to cope with complex combinations of stresses. Regulator genes act as switching devices, facilitating these switches. Stress response networks exhibit a modular structure, where different modules generate responses to various stresses^35^. Then, the regulators switch between these modules. In our paper, however, we do not delve into these questions regarding the structure of networks. We simply utilize known estimates of the accuracy of approximation of target functions via Deep Networks^36–38^. These estimates describe the dependence of approximation accuracy on the network size $N_{reg}$, but it is challenging to infer details about the network structure, except for the fact that the network is deep and the vertices have a small degree.

Furthermore, we demonstrate the versatility of this function as it is capable of describing not only a free-living bacterium but also various cooperation phenomena. Our findings can be summarized as follows: a new form of cooperation emerges when the computational cost of cooperation is lower than that of direct adaptation without cooperation. To illustrate this concept, we explore the evolutionary adaptivity barriers in the in *silico models* labeled **A–D**.

We initially examine the evolutionary adaptivity barriers for various models without cooperation (the case **A**). This entails investigating whether well-adapted phenotypes exist for individual organisms without engaging in cooperation. In this case, the fitness maximum is achieved if all $f_i=1$. By making certain assumptions, such as the randomness of genetic regulation (as described in Assumption **RG** in the SM), we derive the following results.

The adaptivity barrier can be expressed via the quantity $P_{+, \delta }$, which is a maximal probability to find a constraint *i* and the gene expression string *s* such that $f_i(s)>1-\delta$:

2.3 $$\begin{aligned} P_{+, \delta } =\max _{i \in [1, M], \ s \in S^N} \Pr [ f_i (s, \textbf{w}) > 1-\delta ], \end{aligned}$$

where $\delta \in [0,1)$ is a small parameter, $[1,M]=\{1,2,..., M\}$ and $S^N=\{0, 1\}^N$ the set of all genotypes.

If $P_{+, \delta }$ is not small there exists a genotype *s* satisfying conditions $f_i(s)>1-\delta$ for all $i=1,..., M$, i.e., a genotype sufficiently well adapted with respect to all *M* constraints. We find the following upper bound for *M*:

2.4 $$\begin{aligned} M < C_{\textrm{max}} N, \quad C_{\textrm{max}} = \frac{\ln 2}{|\ln P_{+,\delta }|}, \end{aligned}$$

where *N* is the number of genes, and the constant $C_{\textrm{max}}$ is uniform in *M* and *N* as both $M, N \gg 1$. If condition (2.4) is not satisfied, the likelihood of a well-adapted phenotype becomes highly improbable, with its probability exponentially approaching zero (as detailed in Proposition I from the SM). Therefore, inequality (2.4) acts as a constraint on the maximum adaptivity achievable by an organism without cellular cooperation. The specific values of $C_{\textrm{max}}$ for various models are discussed in depth in the SM. We focus on two basic models here: the *K*-SAT and single-layered perceptrons (SLPs), as used in^20^. The *K*-satisfiability problem (*K*-SAT), a fundamental and challenging combinatorial problem^10^, has a clear biological interpretation. Biologically, it represents *M* constraints, each dependent on *K* Boolean genes, which must be satisfied by the correct expression of *K* genes involved. The number of satisfied constraints can be viewed as the fitness of a Boolean string of length $N>> K$. For large *K*, one finds that $C_{\textrm{max}} \approx 2^K \ln 2$. However, if the number of genes involved in each trait is randomly distributed according to a Poisson distribution with a mean of *K*, then $C_{\textrm{max}} \approx \exp (K/2) \ln 2$, indicating that randomization reduces the barrier. It is also important to note that gene redundancy, as defined by the parameter *K*, significantly increases the adaptivity barrier and, consequently, adaptability.

The SLP model is defined by

2.5 $$\begin{aligned} f_j =\sigma \left( \sum _{i=1}^{N} w_{ji}{s_i} - h_j\right) , \end{aligned}$$

where $j = 1, \ldots , M$. Here $\sigma (z)$ is a sigmoidal function of *z* such that $\sigma$ is monotone increasing, $\sigma (z) \rightarrow 1$ as $z \rightarrow +\infty$ and $\sigma (z) \rightarrow 0$ as $z \rightarrow -\infty$. The coefficients $w_{ij}$ can have different signs. If $w_{ji}>0$ then the *i*-th gene is an activator for *j*-th trait, if $w_{ji}<0$ it is a repressor, and for $w_{ji}=0$ that gene does not affect the *i*-th trait. This model can be viewed as a circuit with the ability to regulate phenotype robustness through special parameters $h_j$, which are thresholds first introduced in^39^. The model as described in equation (2.5) has been considered in^40–42^.

Suppose that all $w_{ij}$ are independent, normally distributed, random quantities with zero mean and variance $r^2$. These assumptions, which are standard for such models^43^, imply that we are considering a random gene regulation. Then

2.6 $$\begin{aligned} C_{\textrm{max}} =\ln 2 (\ln {2\pi }^{-1/2} \int _{{\bar{h}}}^{\infty } \exp ( - u^2 /2) du)^{-1}, \end{aligned}$$

where the quantity ${\bar{h}}= h (\sqrt{N } r)^{-1}.$ can be interpreted as a normalized threshold, and it defines the sensitivity of the trait $f_i$ with respect to mutations.

It is noteworthy that in multilayered models, the coefficient $C_{\textrm{max}}$ may substantially exceed that of single-layer models. This suggests that cascade regulation can alleviate the complexity cost, as detailed in the SM.

In case **B**, we examine a symbiotic relationship involving *n* organisms using the Set Covering Model (see the SM for more details). We define $N_{reg}$ as the number of genes in the set $S^{reg}$ required to facilitate cooperation or symbiosis. For instance, in the context of symbiosis transitioning from prokaryotes to eukaryotes, these genes $S^{reg}$ may be responsible for the adhesion of different organelles, such as mitochondria. The transition from case **A** to case **B** becomes computationally advantageous when the following condition is met: $N_{reg} < C_{\textrm{max}}^{-1} \frac{(n-1)M}{n}.$ It is reasonable to expect that this inequality holds true in challenging environments characterized by a large number of potential ecological constraints ($M \gg 1$).

**Figure 2:**
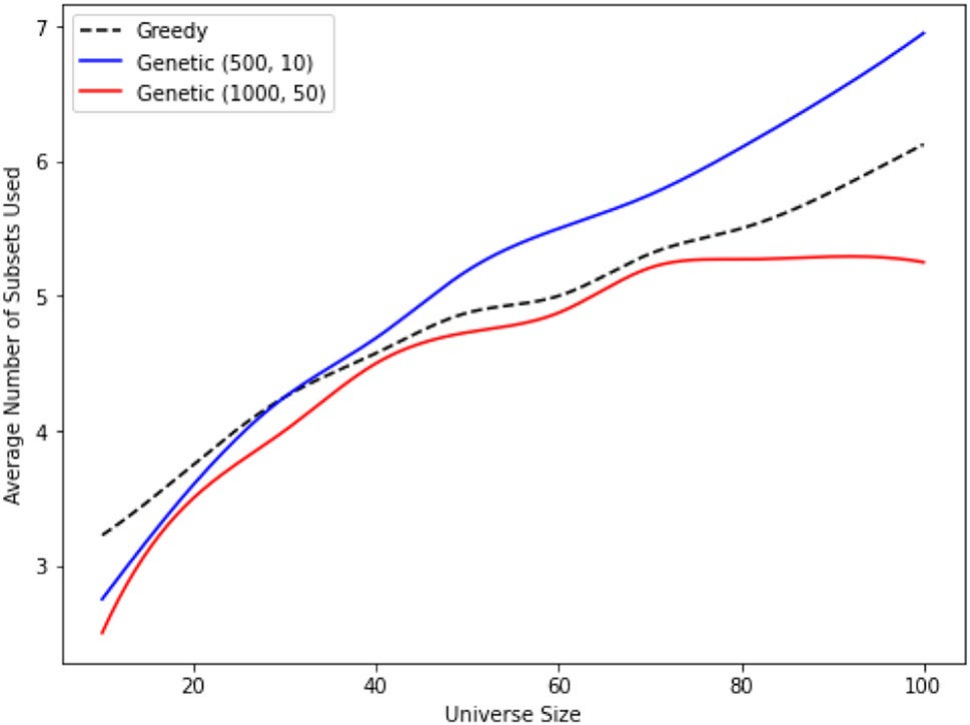
The plot illustrates the resource usage by genetic and greedy algorithms. Mathematically, resources are represented by the number of subsets, denoted as $N_{sub}$, required to cover a given set. The comparison is made between the greedy algorithm and various simulations of a genetic algorithm, with each simulation defined by two parameters (e.g., (1000, 50) indicating 1000 steps with populations of 50 members). The plot demonstrates that the most sophisticated genetic algorithm achieves the task of covering the set using the least value of $N_{sub}$, indicating efficient resource utilization.

Consider the Jacob-Monod regulation^44^. In the Monod–Jacob model, the *Escherichia coli* lac operon encodes proteins necessary for the transport and breakdown of the sugar lactose (lac). The production of proteins is prevented when a repressor, encoded by a regulatory gene, binds to its operator, a specific site in the DNA sequence that is close to the genes encoding the proteins. If each organism possesses a sensor to detect which products are already produced by other organisms, it eliminates the need to produce those products. Maximizing fitness, therefore, involves producing the maximum number of products that are not already synthesized by other organisms. Furthermore, it is important to note that this regulation minimizes energy consumption, as discussed in^45^. The Jacob–Monod regulation is limited to systems whose functioning does not rely on feedback loops composed of the gene regulatory system and external input. Mathematically, this regulation can be interpreted as a greedy algorithm, as outlined in the SM. The greedy approach implies that each organism aims to satisfy constraints that remain unsatisfied by others, thus maximizing its individual fitness (Fig. 2). Let us estimate $N_{reg}$ for this greedy algorithm.

Note that, in order to perform this regulation, the *i*-th cell must have sensors capable of recognizing $$M \theta _i$$ different reagents, where $$\theta _i$$ is on the order of 1/*n*. Therefore, it can be expected that in this case, $$N_{reg} $$ is on the order of *M*/*n*. Consequently, for the minimal required gene number *N*, we obtain $$ N > C_{reg} \frac{M}{n}=N_{reg}, $$ where $$C_{JM}$$ is a constant $$>1$$ while for individual cells we have $$N = M>> N_{reg}$$. So, the transition from **B** to **C** is computationally profitable if the parameter *n* satisfies $$ n> C_{reg}$$. This is attributable to the fact that a single regulatory gene can coordinate the expression of multiple proteins.

Furthermore, for the cases **A**, **B**, and **C**, as well as the fitness functions examined in^20^, the search for a gene string *s* that maximizes the fitness can be reduced to an NP-hard Boolean linear programming problem. However, in the transition from case **C** to case **D**, as demonstrated in the SM, our model indicates that in multicellular organisms, the search for a response to stress leads to a standard real-valued linear programming problem. In algorithm theory, it is well-known that transitioning from discrete to real variables significantly simplifies the problem (a process known as relaxation). In our case, these real variables represent the concentrations of cells of a given type. Unlike the NP-hard problem, this real-valued linear programming problem can be feasibly solved within a reasonable timeframe. Such a property potentially gives multicellular organisms a decisive advantage, as their genetic networks can find a response to stress much faster and have higher chances of success.

Transitions from simple symbiotic systems to cell colonies can occur in response to a decrease in available resources. Consequently, colonies of cells with the Jacob–Monod regulation demonstrate greater adaptability to the environment compared to ensembles of non-interacting cells. This increased adaptability arises from the fact that cell colonies consume fewer resources (see Fig 2).

The scarcity of resources available for consumption may also provide an explanation for the transition from cell colonies to more complex multicellular organisms with gene regulation. We exploit the interpretation of simple gene regulations as greedy algorithms, which, in general, is not optimal and cannot achieve the optimal value of the target function. In our case, the target function is the number of consumed resources, as indicated by relation (4.8). If the critical constant $C_{res}$, representing the maximum level of available resources, falls below the value attainable by the greedy algorithm, the simple cell colony encounters an extinction challenge. Therefore, the transition from simple cell colonies to multicellular organisms, where more sophisticated gene regulation algorithms are at play, can be attributed to the need to overcome starvation and enhance resource utilization. The transition to higher forms through cooperative evolution does not necessarily result in the complete extinction of lower forms. In fact, it is known that the current distribution of life’s kingdoms is as follows: plants dominate with approximately 450 gigatons of carbon (Gt C), while animals account for about 2 Gt C, bacteria for approximately 70 Gt C, and archaea for around 7 Gt C^46^. Microorganisms forming simple colonies can benefit from symbiosis with more complex forms. For example, mammalian intestines provide relatively stable environments for bacteria^47^.

## Discussion

The emergence and evolution of complexity are fundamental challenges in biology^1,2,48^. It is intriguing to consider why the evolution of life did not cease at the stage of the simplest autotrophic prokaryotes but produced “organisms with huge, regulated genomes, multiple tissue types, and even ability to develop theories of evolution?” (see^48^, Ch. 8).

This manuscript demonstrates that classical models from algorithm theory and mathematical methods from statistical mechanics, originally developed for neural networks, spin glasses, and Boolean satisfaction problems, can provide insights into the emergence of new forms of cooperation in life. Inspired by^5,6^, we view evolution as a hard combinatorial problem and draw upon the foundational principles of algorithm theory^10,21^. The key concept, as highlighted in^5,6^, is that evolution is achievable if the time required for adaptation is polynomial in the number of genes, but it becomes infeasible if this time is exponentially large. Notably, the existence of a fundamental adaptivity barrier was first identified by R. Fisher in the context of the Fisher Geometric model^18^, and subsequent studies by Orr^19,34^ further explored this concept. In this manuscript, we also demonstrate that such an adaptivity barrier is present in practically all Boolean fitness models, which encompass gene-trait maps and generalize the Fisher model with mutations that have been extensively studied^49^.

A second important idea, proposed in^31^, suggests that all metabolic systems with fixed parameters are inherently unstable under fluctuations and will eventually be destroyed. In order to maintain stability and avoid destruction, evolution needs the process of replication, which involves the invention of new genes and modifications to existing ones. This concept was explored further in^50^, leading to fundamental implications. The introduction of new genes requires cells to develop more complex metabolic systems, thereby giving rise to a generator of complexity. Consequently, the evolution of individual organisms inevitably encounters a fundamental adaptivity barrier. This barrier hinders gradual evolution, prompting the emergence of new forms of cooperation when the computational (and thus thermodynamic) cost of gradual evolution becomes too high to overcome the adaptivity barrier.

Complex traits can evolve adaptively or non-adaptively. The debate over the nature of evolution has been ongoing for many years, starting with Kimura’s seminal work^51^ and continuing with others like^48^. Genomic data, coupled with the relatively small effective population sizes of large multicellular organisms, support the opinion that the evolution of these organisms might have been non-adaptive, aligning with the theory of constructive neutral evolution. Based on our models, drawing a definitive conclusion remains challenging. However, our estimates suggest that the emergence of multicellular organisms was quite plausible.

Undoubtedly, constructing an efficient genetic network is a complex process. Nevertheless, our estimates indicate that even a slight increase in network size can exponentially enhance the probability of maintaining homeostasis, provided the initial GRN was sufficiently large. The likelihood of a series of mutations leading to a more efficient network is small, but not exponentially so, in terms of the network size parameter $N_{reg}$. Our calculations reveal that even if almost all mutations were non-adaptive, except for the final terminal mutation, the process of successive replications leading to an organism with complex genetic regulation is not implausibly rare.

Using the Boolean models, we can demonstrate that certain forms of cooperation may arise as a result of rapid environmental changes. When faced with bounded mutation rates and a limited supply of genes within individual organisms, it becomes insufficient to adapt to new and challenging environmental constraints. In such cases, cooperation between organisms becomes the only viable option for survival.

Furthermore, some forms of cooperation emerge due to resource limitations. For example, under inappropriate environmental conditions but with abundant nutrients, slime mold exists as a colony of single cells. However, when faced with harsher conditions such as starvation, the cells undergo migration, aggregation, and differentiation into spores. This transformation from colonies to a primitive multicellular organism aligns with our model for the transition from case **C** to case **D**.

The algorithmic approach highlights that the biosphere is not capable of adapting to rapid environmental changes within short periods. As a result, these rapid changes can lead to the emergence of new cooperative forms or mass extinctions. A notable example of this is the Cambrian explosion, a period associated with global warming caused by the greenhouse effect^52^. During this period, there was a substantial increase in biodiversity and the emergence of multicellular organisms. The exact reasons for these developments are still subject to debate, but one leading theory suggests that the increase in atmospheric oxygen levels played a crucial role. The higher oxygen levels allowed cells to adhere together and form complex body structures, and they also facilitated metabolic processes involved in the production of collagen, a protein critical for the formation of hard body structures.

On the contrary, there are theoretical arguments and comparative genomics data that support the second scenario, known as the neutral or non-adaptive scenario^53^. This perspective, discussed in detail in^48^ (Chapters 8-9), suggests that random evolutionary processes and genetic drift play an important role in the emergence of large, complex genomes, rather than solely adaptive selection.

Our theoretical arguments align, to some extent, with this non-adaptive theory. The process of random evolution leading to the emergence of large, effective regulation networks of size *N* may require a number of steps, most of which do not contribute to an immediate increase in fitness. However, once this network is established, it provides a substantial fitness advantage (exponential in $N^{\gamma }$) that facilitates the fixation of complex regulated genomes, even in small populations.

## Methods

Our approach is built upon several established components: (**i**) The concept of replicative stability in systems supporting homeostasis, as proposed Gromov-A.Carbone^31^; (**ii**) The fitness model developed Reinitz et al.^20,41^; (**iii**) The theory of large deviations and stochastic transitions in random dynamical systems^54^; (**iv**) The theory of phase transitions in combinatorial problems^21^; (**v**) The novel idea employed in this study is the stability via gene regulation, as described in^25^ and the SM. In this context, we use universal approximations provided by deep networks^36–38,55^.

The application of these methods can be described as follows. We use (**i), (ii), (v)** to estimate the stochastic stability of evolving biochemical systems under random perturbations. Note that the fundamental results of large deviation theory, as outlined in point **(iii)**, are applied to estimate the probability that the organism will remain within the homeostasis zone and survive.

This analysis leads us to the development of combinatorial fitness models, which have the ability to describe both individual organisms and various cooperative communities. Furthermore, with the help of (**iv**), we estimate the adaptivity barriers associated with different situations. In the subsequent subsections, we will introduce the specific models that are used.

### Metabolic regulation and homeostasis

Here, we briefly describe a GRN model and homeostasis. This model is also described in more detail in^25^ and the SM.

Let $v_1,..., v_n$ represent the concentrations of chemical reagents involved in the metabolism of an organism. Biochemical kinetics can be described by the following system of differential equations:

4.1 $$\begin{aligned} \frac{dv_i}{dt}= { g_i}( v, \xi , s), \quad t \ge 0, \end{aligned}$$

where $v=(v_1, ...., v_n) \in D$, where *D* is a compact domain in a non-negative cone ${{\mathbb {R}}}_{>}^n=\{v \in {{\mathbb {R}}}^n: \ v_i \ge 0 \}$ with a smooth boundary ${\partial D}$, $v_i(t)$ are the concentrations of reagents, the reaction terms $g_i$ are smooth functions, for example, polynomials (see the SM), in *v* dependent on $\xi$, which is a random stress parameter, and the gene expression string *s*. Reaction rates $g_i$ depend on the state of the random environment, characterized by the coordinate $\xi$ in ${\mathcal {E}} \subset {\mathbb {R}}^d$. Here, ${\mathcal {E}}$ is a set of all possible environments. For instance, $\xi$ could be the concentrations of nutrients necessary for the organism’s survival, the temperature, or parameters describing a complex temperature regime, including variations during different times of the day.

To incorporate genes into our models, we use Boolean strings $s = (s_1, ..., s_{N_g})$, where $N_g$ is the number of genes. Each gene $s_i$ can take Boolean values $s_i \in \{0, 1 \}$.

*Homeostasis.* We assume, to be viable, the organisms should produce sufficiently large amounts of the output concentrations $v_i$: $v_i(\xi ,s) > h_i, i \in I,$ where $I \subset \{1, ..., N_{r} \}$ is a subset of indices. These conditions define the homeostasis (viability) domain, where organisms survive (see the SM). The probability $P_{v}(s)$ that the organism remains viable is

4.2 $$\begin{aligned} P_{v}(s) =Pr [ v_i(\xi ,s) > h_i \quad \forall i \in I ]. \end{aligned}$$

Let the random quantity $\xi$ be distributed according to the probability measure $d\rho (\xi )$. Then

4.3 $$\begin{aligned} P_{v}(s) =\int _{V(h, N_r,s)} d\rho (\xi ), \end{aligned}$$

where the set *V* is defined by $V(h, N_r, s)= \{\xi \in {\mathcal E} : \quad v_i(\xi ,s) > h_i \quad \forall i \in I \}.$ The quantity $P_v(s)$ can be considered as the fitness of an individual. Computing this fitness for real metabolic networks is a challenging task. However, in many cases, it can be shown that minimizing the probability $P_v(s)$ reduces to solving different Boolean combinatorial problems, which are described in the following subsections (refer to the SM for more details).

The quantity $P_v(s)$ can be considered as the fitness of an individual. Computing this fitness for real metabolic networks presents a challenging task. However, in many cases, it can be shown that minimizing the probability $P_v(s)$ is equivalent to solving various Boolean combinatorial problems, which are described in the following subsections (refer to the SM for more details).

*GRN.* One can show (see the SM), through the Fourier analysis of pseudo-Boolean functions, that the kinetic rates $g_i$ can be split into two parts: $g_i^{pert}$, interpreted as a perturbation induced by the stress $\xi$, and $g_i^{reg}$, which depends on gene expression. The second part can be considered as a GRN. We can ensure the stability of the system under perturbations if the perturbation and the response to it mutually negate (or almost negate) each other.

As an example, let us consider mechanisms that allow bacteria to resist antibiotics. Bacteria have different methods to withstand the effects of an antibiotic. For instance, some bacterial enzymes can inactivate antibiotics. An example can be given by $\beta$-lactamase, which destroys the active component of penicillins. Bacteria can also produce enzymes capable of adding different chemical groups to antibiotics, preventing the binding between the antibiotic and its target in the cell. This resistance mechanism can be described by our model.

#### Simplest fitness model

This model (as described in^20,32^) can be obtained by considering a chain of reactions following the Michaelis–Menten law, where each stage is controlled by a gene. In this context, we can examine the gene-trait maps defined by

4.4 $$\begin{aligned} f_j =\sigma \left( \sum _{i=1}^{N} w_{ji}{s_i} - h_j\right) , \end{aligned}$$

where $j = 1, \ldots , M$. Here $\sigma (z)$ is a sigmoidal function of *z* such that $\sigma$ is monotone increasing, $\sigma (z) \rightarrow 1$ as $z \rightarrow +\infty$ and $\sigma (z) \rightarrow 0$ as $z \rightarrow -\infty$. The coefficients $w_{ij}$ can have different signs. If $w_{ji}>0$ then the *i*-th gene is an activator for *j*-th trait, if $w_{ji}<0$ it is a repressor, and for $w_{ji}=0$ that gene does not affect the *j*-th trait. This model can be viewed as a circuit that allows for the regulation of phenotype robustness through specific parameters $h_j$, which are thresholds initially introduced in^39^. Similar models, such as (2.5), have also been explored in studies such as^40–42^.

Suppose that all $w_{ij}$ are independent and normally distributed random variables with zero mean and variance $r^2$. These assumptions, which are standard for such models^43^, imply that we are considering random gene regulation.

#### Multilayered perceptron (MLP)

To see that the MLP can appear as a model of metabolism we can consider two metabolic ways, which work in a cooperative way (see the SM).

MLPs (Multi-Layer Perceptrons) typically consist of one or several hidden layers arranged in a feed-forward cascade. In each layer, the genes depend recursively on the genes in the previous layer through sigmoidal functions. This architecture resembles the gene regulatory networks observed in developmental biology, such as the cascade of transcription factors during *Drosophila melanogaster* morphogenesis. Maternal factors activate gap or pair-rule genes, which in turn activate Hox genes, and Hox genes activate realizator genes that drive segment differentiation in the developing embryo. Therefore, MLP architectures can effectively mimic realistic gene regulatory networks encountered in developmental biology. Furthermore, MLP architectures are Turing-complete, meaning they have the ability to approximate any Boolean function, including any conceivable gene-trait map^56^. This versatility allows MLPs to serve as models for various biological processes, including metabolism. For example, by considering two metabolic pathways that operate cooperatively, we can demonstrate how MLPs can effectively represent such interactions (see the SM for more details).

It has been demonstrated that both single-layer and multilayer perceptron models are capable of reproducing the topological characteristics of rugged and fragmented fitness landscapes observed in real-world situations. This is achieved through the reduction of single-layer perceptron models to the well-known hard combinatorial model called *K*-SAT (see the SM for further details).

### Models of cooperation

All the cooperative models discussed in this context can be derived from the fitness functions described earlier. The fitness functions capture the essential dynamics and interactions within the systems, allowing us to model and analyze various cooperative phenomena.

#### Models for symbiosis

We consider a case where organisms $O_1, O_2,..., O_m$ coexist in an external environment. These organisms have the ability to activate different genotypes $s^{(1)}, s^{(2)},..., s^{(n)}$ that enable them to produce chemical compounds (gene products) essential for their survival. Let us denote by $a_{il}$ the quantity of *i*-th gene product that can be obtained by activating genotype $s^{(l)}$. The cost of activating genotype $s^{(l)}$ is denoted by $r_l$, which represents the amount of resources required. Additionally, $h_i$ represents the minimum amount of the *i*-th gene product needed for the survival of the organism. Mathematically, the cooperation problem among organisms can be formulated as an Integer Linear Programming problem, which can be defined as follows: *To minimize*

4.5 $$\begin{aligned} F_{targ}(u)= \sum _{l=1}^n r_{l} u_l \end{aligned}$$

*under conditions*

4.6 $$\begin{aligned} \sum _{l=1}^n a_{il} u_l \ge h_i, \quad \forall i=1,..., M \end{aligned}$$

4.7 $$\begin{aligned} u_l \in \{0,1\}. \end{aligned}$$

This means that each organism produces a subset of gene products, and collectively they need to produce all the products required for survival using the minimal amount of resources defined by $F_{targ}(u)$. The Boolean variable $u_l$ represents the activation status of the genotype $s^{(l)}$, where $u_l=1$ if the genotype is activated and $u_l=0$ otherwise. It is worth noting that an alternative formulation of the problem can be considered, where the condition (4.5) is replaced by:

4.8 $$\begin{aligned} F_{targ}(u) < C_{res}. \end{aligned}$$

This implies that the population of organisms must adapt and survive under constrained resource availability. In the specific case where $a_{il} \in {0,1}$, $r_l=1$, and $h_i=1$, the Integer Linear Programming problem reduces to the Set Cover Problem^57^. Let’s delve into the Set Cover Problem and its biological interpretation in more detail.

Let $\xi$ represent a parameter that characterizes the state of the environment. We assume the existence of a set ${\mathcal U}={f_{1},..., { f}_{M}}$, which encompasses all possible ecological constraints (referred to as the Universe). For each specific $\xi$, a different subset of constraints ${{\mathcal {E}}} (\xi )\subset {{\mathcal {U}}}$ is present.

Let ${{\mathcal {F}}}$ be a family that comprises *n* subsets ${\mathcal E}_k$, where $k=1,...,n$, representing the different constraints from the Universe ${{\mathcal {U}}}$. We assume that the *k*-th organism has the ability to fulfill the constraints specified by a set ${{\mathcal {E}}}_k \in {{\mathcal {F}}}$ from this family.

We suppose that

4.9 $$\begin{aligned} {{\mathcal {U}}}={ \bigcup }_{k=1}^n {{\mathcal {E}}}_{k}, \end{aligned}$$

i.e., all the sets ${{\mathcal {E}}}_{k}$ cover the entire Universe ${{\mathcal {U}}}$.

Consider different subfamilies ${{\mathcal {E}}}_j \subset {{\mathcal {F}}}, j \in J$, where *J* is a subset of $[1,n]=\{1,..., n\}$. Let us denote by |*J*| the number of elements of the set *J*, i.e., the size of the subfamily. Then the Set Covering Problem is as follows:

*Consider the family* ${{\mathcal {F}}}$ *of subsets of* ${{\mathcal {U}}}$. *Suppose* (4.9) *is satisfied. For a subset* ${{\mathcal {E}}} \subset {{\mathcal {U}}}$ *to find a cover* ${{\mathcal {E}}}_j$ *of* ${{\mathcal {E}}}$ *by subsets* ${{\mathcal {E}}}_j \in J$:

4.10 $$\begin{aligned} {{\mathcal {E}}} \subset \bigcup _{j \in J} {{\mathcal {E}}}_j, \end{aligned}$$

*that has a minimal size* |*J*|. In other words, we have a set ${{\mathcal {U}}}$ containing elements 1, 2, ..., *M*, referred to as the Universe, and a collection ${{\mathcal {F}}}$ consisting of *m* sets. The objective is to find the smallest subset of ${{\mathcal {F}}}$ such that the union of this subset covers the entire Universe ${\mathcal U}$ (note that there may be multiple possible subsets that satisfy this condition).

### Supplementary Information

Supplementary Information.

## Data Availability

All data generated or analyzed during this study are included in this published article and its supplementary information files. The code to produce numerical results is available at https://doi.org/10.5281/zenodo.6481568.
