# Supplementary material for: Evolution of biological cooperation: an algorithmic approach
Source: Sci Rep. 2024 Jan 17;14:1468. doi: 10.1038/s41598-024-52028-0 (PMC10794236; doi:10.1038/s41598-024-52028-0)
Supplement: Supplementary file 1 — Supplementary Information. [file 41598_2024_52028_MOESM1_ESM.pdf]

Supplementary material to the manuscript "Evolution of biological cooperation: an algorithmic approach"
by *I. Sudakow, J. Reinitz, S. Vakulenko, D. Grigoriev* submitted to Scientific Reports, 2023

The material is organized as follows: In Section 0.1, we present kinetic models, detail how genes are incorporated into these models, and define system viability under stochastic perturbations. In the subsequent Section 0.2, we describe some preliminaries: transforming kinetic equations to account for gene regulation in response to stress perturbations. The main new ideas are presented in Sections 0.3 and 0.4, focusing on replicative stability and the application of Freidlin–Wentzell theory to estimate this stability. Furthermore, we examine the complexity barriers for cases **A** to **D**.

0.1 Models and viability

0.1.1 Chemical kinetics

Some Hard Combinatorial problems appear, in a natural way, when we consider how genes support homeostasis. Let $v_1, ..., v_{N_r}$ be concentrations of chemical reagents involved in the metabolism of an organism (for example, a bacterium). These concentrations v_j depend on a state of random environment $\xi \in \mathcal{E} \subset \mathbb{R}^d$, where $\mathcal{E}$ is a set of all possible environment states. For example, ξ can be concentrations of nutrients needed for that organism's survival, temperature, or parameters describing a complicated temperature regime (temperature at night, morning, and daily temperature).

Systems of differential equations to describe chemical kinetics equations have the form

$$\frac{dv}{dt} = g(v, \xi), \quad t \geq 0, \tag{A1}$$

where $v = (v_1, ..., v_n) \in D$, where D is a compact domain in a non-negative cone $\mathbb{R}^n_> = \{v \in \mathbb{R}^n : \ v_i \geq 0\}$ with a smooth boundary ∂D, $n = N_r$ is the number of reagents, $v_i(t)$ are reagent concentrations, the reaction terms g_i are sufficiently smooth functions, for example, polynomials in v depending on ξ, where a ξ is a random stress parameter.

In the standard models of chemical kinetics, one uses a polynomial model, which can be derived from the law of mass action:

$$g_i(v, \xi) = \sum_{a \in \mathcal{R}_i} C_{i,a}(\xi) v^a, \quad i = 1, 2, ..., N_r \tag{A2}$$

where

$$v^a = v_1^{a_{i1}} v_2^{a_{i2}} ... v_n^{a_{in}},$$

$a = (a_1, ..., a_n)$ is a multiindex with integers $a_i \geq 0$, $C_{i,a}$ are coefficients that determine kinetic rates and $\mathcal{R}_i$ is a set of reactions involved in the production-degradation of i-th reagent. Other popular models involve rational in v nonlinearities, for example,

$$g_i(v) = \sum_{a \in \mathcal{R}_i} C_{i,a} P_{i,a}(v)/Q_{i,a}(v), \tag{A3}$$

where $P_{i,a}(v)$ and $Q_{i,a}(v)$ are polynomials. A typical example of the dependence (A3) is given by the Michaelis–Menten law:

$$g_i(v_j) = \frac{b_i v_j}{a_i + v_j}, \tag{A4}$$

where v_j is an abundance of the input substrate. In more complicated situations we can have a set of substrates, for example, the Michaelis–Menten reaction rate with two input substrates, v_j and v_l, has the form:

$$f_i(v_j, v_l) = \frac{b_i v_j v_l}{a_i + a_{i,1} v_1 + a_{i,l} v_l + a_{i,lj} v_j v_l}. \tag{A5}$$

To incorporate genes in our models, we use Boolean strings. Let $s = (s_1, ..., s_N)$ be a gene expression string. We have N genes s_i, which take Boolean values $s_i \in \{0, 1\}$. The i-th gene may be switched on (when $s_i = 1$) or switched off (when $s_i = 0$). We suppose that all kinetic rates in (A1) can depend on these genes s_i, where gene string $s \in \mathcal{S}^N$, $\mathcal{S} = \{0, 1\}$. The reaction parameters are regulated by the genotype and affected by environmental parameters (for example, temperature). For example, in the case of the Michaelis–Menten dynamics, all parameters depend on ξ and s: $b_i = b_i(s, \xi), a_i = a_i(s, \xi), a_{i,j} = a_{i,j}(s, \xi)$.

0.1.2 Viability in simplest cases

We assume, to be viable, the organisms should produce sufficiently large amounts of the output concentrations v_i:

$$v_i(\xi, s) > h_i, \quad i \in I, \tag{A6}$$

where $I \subset \{1, ..., N_r\}$ is a subset of indices. Conditions (A6) define the homeostasis (viability) domain, where organisms survive. The details of biochemical dynamics are unimportant to us, it may be stationary in time, or periodic dynamics. If the dynamics is time-periodic and v_i depends on time t we take averages over a cycle period. If the dynamics produces a time stationary regime, we suppose that v_i are equilibrium concentrations.

The probability $P_v(s)$ that the organism remains viable is

$$P_v(s) = Pr[v_i(\xi, s) > h_i \quad \forall i \in I]. \tag{A7}$$

Let the random quantity ξ be distributed according to probability measure $d\rho(\xi)$. Then

$$P_v(s) = \int_{V(h,n,s)} d\rho(\xi), \tag{A8}$$

where the set V is defined by

$$V(h,n,s) = \{\xi \in \mathcal{E} : \quad v_i(\xi,s) > h_i \quad \forall i \in I\}.$$

The quantity $P_v(s)$ can be considered as a fitness of an individual. For real existing metabolic networks, computing this fitness is a formidable task. In fact, even for *Escherichia coli*, such networks involve thousands of reactions, genes, and gene products. However, we will find certain approximations under some simplifying assumptions, which are nonetheless natural from a biochemical standpoint.

0.1.3 Example: Viability for simple metabolic chains

Let us suppose that different products v_j can be produced via M independent different metabolic ways going in a few stages, where each stage is defined by the Michaelis–Menten law (A4). At each stage, there also exists a degradation process. We then have:

$$\frac{dv_{i,k}}{dt} = \frac{b_{i,k}(s)v_{i,k-1}}{a_{i,k}(s) + v_{i,k-1}} - \lambda_{i,k}(s)v_{i,k}, \tag{A9}$$

where i indicates the way number, $k = 1, 2, ..., K_i$ stands for the stage index, K_i is the i-th way length, $\lambda_{i,k}$ are degradation coefficients, $b_{i,k}$ and $a_{i,k}$ kinetic coefficients, $v_{i,0} = R_i$ are random input resources and $v_i^{out} = v_{i,K_i}$. We assume that input concentrations R_i evolve in time slowly so we can apply a quasi-stationary approach. Then by an induction over k one has

$$v_i^{out} = \frac{\bar{b}_i(s)R_i}{\bar{a}_i(s) + R_i} + s.c., \tag{A10}$$

where $\bar{b}_i, \bar{a}_i$ are coefficients, which can be expressed via $\lambda_{i,k}$, $b_{i,k}$ and $a_{i,k}$, and $s.c.$ denotes a small corrections. If concentrations R_i are small

$$v_i^{out} \approx \frac{b_{i,1}(s)b_{i,2}(s)...b_{i,K_i}(s)}{a_{i,1}(s)\bar{\lambda}_{i,1}(s)...a_{i,K_i}(s)\bar{\lambda}_{i,K_i}(s)}. \tag{A11}$$

For the case, where all resources R_i are independent, one obtains

$$P_v(s) = F(s) = \prod_{j=1}^{M} f_j(s), \tag{A12}$$

where

$$f_j(s) = \Pr\left[R_i > \frac{\bar{a}_i(s)h_i}{\bar{b}_i(s) - h_i}\right]$$

and $P_v(s) = F(s)$ can be considered as a fitness.

0.1.4 Environment impact

In general case, each f_i is a complicated function of Boolean arguments. If we take into account a dependence on environment parameters, for example, temperature T, (we know that kinetic parameters are very sensitive with respect to temperature) one obtains

$$f_j(s,T) = \Pr\Big[R_i > \frac{\bar{a}_i(s,T)h_i}{\bar{b}_i(s,T) - h_i}\Big].$$

If we take into account not only the average temperature values but also its variations (weather, for example, weather changes during the day), we can get more complex dependencies. Consider, for example, eq. (A4), where $b_i = b_i(s,T), K_i = K_i(s,T)$ and T depends on the time t. Then, by solving linear equation (A9) with unknown $v_k(t)$ and a given $v_{k-1}(t)$, one has

$$v_{i,k}(t) = \int_0^t \exp\big(-\lambda_{i,k}(s)(t-\tau)\big)\frac{b_{i,k}(s,T(\tau))v_{i,k-1}(\tau)}{a_{i,k}(s,T(\tau)) + v_{i,k-1}(\tau)}d\tau, \qquad (A13)$$

where we suppose that $v_{i,k}(0) = 0$. To obtain P_v, we must take an averaging over all possible trajectories $T(\tau)$ and all reaction stages. This appears to be an intractable problem; to circumvent this difficulty, we will adopt a more phenomenological approach. In the upcoming subsection, under additional assumptions, we derive a simplified expression for f_i, which renders the problem more mathematically tractable.

0.1.5 Reduction to hard combinatorial problems: the simple cases

Suppose (A11) holds and each reaction stage depends on a single protein-coding gene. Then v_i^{out} is a product of K_i real-valued functions $\alpha_k(s)$, where each factor α_k depends on a single Boolean variable, thus, $\alpha_k = w_{ik}s_{l_{i,k}} + \bar{w}_{ik}$. Then, as a result of straightforward computations, we find the following approximation

$$f_i(s,T) = \Pr\Big[\ln\ R_i > y_i(s,T) = \sum_{j=1}^{N} w_{ij}(T)s_j + h_i(T)\Big], \qquad (A14)$$

where w_{ij}, h_i are coefficients.

This gives us the fitness model defined by (A12) and (A14), where f_j are functions of the Boolean genotype s, $j = 1, \ldots, M$. Note that $f_i \in (0,1)$, that f_i depend on $y_i(s)$ and they have sigmoidal form as functions of y_i. Therefore there occurs a single layer perceptron.

The functions f_j can be interpreted as traits (features) of organisms, that define its viability. A variable s_i occurs explicitly in the formula $f_j(s)$ if the j-th trait is affected by the i-th gene. The map $s \to f_1, f_2, ..., f_M$ is then a gene-trait map.

0.2 Transformation of kinetic equations

A more general approach is developed in this subsection. We start with a transformation of kinetic equations.

0.2.1 Some preliminaries on pseudo-Boolean functions

Real valued functions $F(x)$ of the discrete argument $x \in \{-1, 1\}^N$ (pseudo-Boolean functions) can be represented by an analog of the Fourier decomposition. Namely, every real-valued function $F(x)$ has a unique expansion as a multilinear polynomial [1]:

$$F(x) = \sum_{S \in [N]} \hat{F}(S)\chi_S(x), \tag{A15}$$

where $[N] = \{1, 2, ..., N\}$, the sum is taken over all subsets of $[N]$ and

$$\chi_S(x) = \prod_{i \in S} x_i.$$

Note that this Fourier representation is basic and has many applications in combinatorics, social choice theory, random graphs, and theoretical computer science [1]. We put $F = g_j$ in (A15) and by the variable change $s_i = (1 + x_i)/2,\ x_i = 2s_i - 1$ we find the following representation:

$$g_j(v, s, \xi) = \bar{g}_j(v, \xi) + g_j^{reg}(v, s, \xi), \tag{A16}$$

where g_j^{reg} are Lipshitz in v and ξ. These functions define a regulatory effect of gene expression.

0.2.2 Transformation of kinetic equations

By substituting g_j in the form (A16) into the right hand sides of (A1) we obtain the system

$$\frac{dv_j}{dt} = g_j^{reg}(v, s, \xi) + \bar{g}_j(v, \xi). \tag{A17}$$

This system will be the main object of our research.

To provide existence and uniqueness of solutions to the Cauchy problem defined by (A17) and initial conditions $v(0) = v^0 \in D$, we assume the following. Let

$$\bar{g}(v, \xi) \cdot n(v) \leq 0 \quad \forall v \in \partial D \tag{A18}$$

and

$$g(v, s, \xi) \cdot n(v) \leq 0 \quad \forall v \in \partial D,\ \forall s \in \mathcal{S}^{N_g}, \quad \forall \xi \in \mathcal{K}_{str}, \tag{A19}$$

where $n(v)$ is a unit normal vector outside D at the point $v \in \partial D$. Due to (A18) and (A19) solutions of that Cauchy problem exist and are unique for all $t > 0$.

We can simplify the problem as follows. Suppose that chemical dynamics is always convergent. Namely, let us suppose that for each ξ there exists an

equilibrium solution v^{eq} of a polynomial system of equations, where coefficients of each polynomial depend of ξ and s:

$$f_i(v^{eq}, \xi, s) = 0, \;\; f_i = \bar{g}_i + g_i^{reg}. \tag{A20}$$

Let $d\rho(\xi)$ be a measure on the space of perturbations ξ. We define the homeostasis probability as follows:

$$P_v(s) = \Pr\{v^{eq}(\xi, s) \in D_H\}, \tag{A21}$$

where D_H is a fixed compact homeostasis domain (for example, a ball) in v-space. If we define a characteristic function $\chi_D(v)$ as 1 for $v \in D_H$ and 0 otherwise then

$$P_v(s) = \int_\xi \chi_D(v^{eq}(\xi, s)) d\rho(\xi). \tag{A22}$$

We identify $s \to P_{sur}(s)$ with the gene-fitness map. The maximization of this map values over s is a hard combinatorial problem. Relation (A22) does not take into account possible small noise in $\xi(t)$. In the next section, we describe more general methods permitting estimating P_v in models of chemical kinetics when ξ contains a fluctuating component.

0.3 Replication and Wiener integral over trajectories for the viability probability

We suppose following Gromov-Carbone [2] that the homeostasis breakdown is a result of small fluctuations. Then we can apply the theory of large deviations for dynamical systems [3] to take into account the effect of these fluctuations.

Let $[0, \tau_r]$ be the generation time period (from a bacterium or cell birthday up to its death or division moment). We can generalize relation (A22) taking into account that the probability P_{out} to leave the homeostasis domain D depends on whole process $\xi(t)$, $t \in [0, \tau_r]$. For each realisation ξ we obtain $P_{out}(\xi(\cdot))$. One can expect that for each stress trajectory $\xi(t)$) the probability $P_{out}(\xi(\cdot))$ can be represented as

$$P_{out}(\xi(\cdot)) = \exp\big(-\beta \varDelta W(s, \xi(\cdot))\big), \tag{A23}$$

where $\beta > 0$ is a large parameter, proportional to the the standard deviation of the noise (the noise magnitude), the $\varDelta W$ is a quantity, which is a potential barrier (see [3]) for gradient like dynamical systems.

Let $d\rho$ be a probabilistic measure on the space of random realizations $\xi(\cdot)$. Then the probability to survive on the interval $[0, \tau_r]$ is

$$P_{v,\tau_r} = 1 - \int \exp\big(-\beta \varDelta W(s, \xi(\cdot)\big) d\rho(\xi(\cdot)). \tag{A24}$$

If we apply the Monte-Carlo method to compute this integral, we then obtain

$$P_{v,\tau_r} \approx 1 - \sum_{k=1}^{M} b_k \exp\big(-\beta \varDelta W_k(s)\big), \tag{A25}$$

where $b_k > 0$ are weight coefficients, $\sum_k b_k = 1$. This relation can be interpreted as follows: we have M variants of external perturbations, k-th variant can be realized with a probability b_k and leads to a system destruction with probability $\exp(-\beta \varDelta W_k(s))$.

To estimate replicative stability, let us consider the probability of survival of a few generations on $[0, T]$, where $T >> \tau_r$. Supposing that for each generation perturbations ξ are independent, we obtain

$$P_{v,T} \approx \prod_{t_0=1}^{N_{ev}} \Big(1 - \sum_{k=1}^{M} b_k \exp\big(-\beta \varDelta W_k(s(t_0))\big)\Big), \quad \text{(A26)}$$

where we take into account that s can mutate at each generation and t_0 is the generation index, $N_{ev} = T/\tau_r$ is the number of generations. It is simpler to compute $\log P_{v,T}$. Then we have

$$\log P_{v,T} \approx \sum_{t=1}^{N_{ev}} \log\Big(1 - \sum_{k=1}^{M} b_k \exp\big(-\beta \varDelta W_k(s(t))\big)\Big).$$

Under the assumption that $\exp\big(-\beta \varDelta W_k(s(t))\big) << 1$ we obtain

$$\log P_{v,T} \approx -\sum_{t=1}^{N_{ev}} \sum_{k=1}^{M} b_k \exp\big(-\beta \varDelta W_k(s(t))\big). \quad \text{(A27)}$$

Let us set

$$f_k(s) \approx 1 - \exp\big(-\beta \varDelta W_k(s)\big). \quad \text{(A28)}$$

Then taking into account that $\sum_{k=1}^{M} b_k = 1$ we obtain

$$\log P_{v,T} \approx \sum_{t=1}^{N_{ev}} F(s(t)), \quad \text{(A29)}$$

where $F(s)$ can be interpreted as a fitness:

$$F(s) = -1 + \sum_{k=1}^{M} b_k f_k(s). \quad \text{(A30)}$$

The next subsection contains a key new idea of our approach.

0.4 Genetic regulation and replicative stability

Here we use some results [4] with some simplifications to show that relation (A27) helps to explain some general evolution principles.

Inspired by the idea of M. Gromov and A. Carbone, they state: 'Homeostasis of an individual cell cannot be stable for a long time as it would be destroyed by random fluctuations within and outside the cell. There is no adequate mathematical formalism to express the intuitively clear idea of replicative stability

of dynamical systems' ([2], p.40). Replicative stability is evidently crucial for the evolution of cancer cells, viruses (such as COVID-19), and bacteria (like E. coli). It's undeniable that replication aids viruses and bacteria in surviving changing environments, as seen in Lensky's experiment with E. coli ([5]).

According to a model in [4], the Gromov-Carbone idea can be formulated in rigorous mathematical terms, and under certain assumptions, this hypothesis is substantiated. The model incorporates results from Artificial Intelligence Theory; specifically, we posit that the response to stress ξ is determined by deep gene networks (DGNs), analogous to deep neural networks (DNNs). This allows us to utilize recent estimates from DNN theory [6, 7, 8, 9].

Suppose

$$\sup_{\xi(\cdot)\in S_\rho} \mathit{\Delta} W(\xi(\cdot)) = \kappa > 0, \tag{A31}$$

where S_ρ is the set of all possible realisations $\xi(t), t \in [0, \tau_r]$, i.e. the support of the measure $d\rho$. This condition means that there is a stress perturbation $\xi(\cdot)$ such that $\mathit{\Delta} W(\xi(\cdot)) > 0$. Under condition (which holds for a large class of systems) we have the following simple result (Theorem 1 from [4]): if the genes are fixed, no mutations and gene regulation is fixed then

$$P_{v,T} < (1-\delta_0)^T, \tag{A32}$$

where $\delta_0 > 0$ depends on the κ.

The most intriguing question is how the expansion of the gene regulation network can stabilize organisms. First, let's consider a useful estimate.

Let us consider a Lipshitz function $g(v)$ defined on a cube $Q = [0,1]^{d_v}$ with the Lipshitz constant ν. We use the explicit estimate from [7]:

$$\epsilon(N_{net}) < 2{d_v}^{1/2}\nu N_{net}^{-2/d_v}, \tag{A33}$$

where ϵ is an approximation accuracy for a network g_{net} of the size N_{net}:

$$\epsilon = \sup_{v\in Q} |g(v) - g_{net}(v)|.$$

Let $N_{reg}(t)$ be the size of the regulation network for generation t. To see the main idea let us consider the stochastic differential equation for $t \in (t_0, t_0+\tau_r)$ (time interval between replications):

$$dv = \bar{g}(v,s)dt + (g(v,s) - g_{reg}(v,s))dW, \tag{A34}$$

where $\bar{g}$ is a determined part, dW is a standard Brownian (Wiener) process, $g(v,s)$ is a perturbation that defines the stress impact and $g_{reg}(v,s)$ be the output of a genetic anti-stress network, which compensates the stress action. The main idea is as follows. Let $N_{reg}(t_0)$ be an increasing sequence of sizes such that the corresponding

$$\epsilon(t_0) = \sup_{v\in Q} |g(v, s(t_0)) - g_{net}(v, s(t_0))| \to 0,$$

then it is possible that

$$P_{v,T} > \delta_1 > 0 \quad \forall T. \tag{A35}$$

In general situation, the problem is quite formidable however we can arrive at interesting conclusions by a rough model. Namely, we suppose that

$$N_{reg}(t_0) = N_0 + p_{inn} t_0,$$

i.e. the network grows in a linear manner and p_{inn} is an innovation rate, which defines the emergence of new units and edges in the gene regulation network. Further, we suppose that v lies in a neighborhood of an equilibrium v^{eq}, replace $g(v, s(t_0)) - g_{net}(v, s(t_0))$ by a constant $\epsilon(t_0)$ and obtain that (A34) transforms into the Ornstein–Uhlenbeck process

$$d\tilde{v} = \mathbf{A}\tilde{v}dt + \epsilon(N_{reg}(t_0))\mathbf{B}dW, \tag{A36}$$

where $\tilde{v}$ is a deviation $v - v^{eq}$ from the equilibrium state, $\mathbf{A}$ is a negatively definite operator with a stable spectrum $Spec\ \mathbf{A}$ such that for $\lambda \in Spec\ \mathbf{A}$ their real parts $Re\ \Lambda < -\delta_{\mathbf{A}} < 0$ and $\mathbf{B}$ is a matrix with the unit norm.

To find a rough estimate of the leaving probabilities P_{out}, let us suppose that the homeostasis domain is the ball D_H centered at the equilibrium v^{eq} of a radius R:

$$D_H = \{||V - v^{eq}|| < R\}.$$

Let $\tilde{v} = v - v^{eq}$. The Ornstein–Uhlenbeck process is a Gaussian process with a zero mean, thus

$$\Pr\{||\tilde{v}|| > R\} = 2(2\pi)^{-1/2}{\sigma_v}^{-1} \int_R^\infty \exp\big(-y^2/2{\sigma_v}^2\big)dy,$$

where $\sigma_v^2 = E||\tilde{v}||^2$. The expected value $E||\tilde{v}||^2$ can be estimated by the fluctuation-dissipation theorem and eq. (A36) (or by Freidlin–Wentzell estimates [3]). We obtain the estimate

$$\sigma_v^2(t_0) = C_{\mathbf{B}}\delta_{\mathbf{A}}^{-1}\epsilon(N_{reg}(t_0))^2, \tag{A37}$$

where a positive constant $C_{\mathbf{B}}$ depends on the operator $\mathbf{B}$ and uniform in $\epsilon > 0$.

For $\sigma_v/R << 1$ we have the following asymptotics

$$\Pr\{||\tilde{v}|| > R\} \approx 2(2\pi)^{-1/2}\sigma_v R^{-1}\exp\big(-R^2/2{\sigma_v}^2\big).$$

Therefore, the probability of leaving the homeostasis domain within the t_0-th generation can be estimated by

$$P_{out}(t_0) \approx \sigma_v(t_0)R^{-1}\exp\big(-C_0 N_{reg}(t_0)^\gamma\big) \tag{A38}$$

where

$$\gamma = 4/N_{str}, \quad C_0 = N_{str}^{-1}(R/\nu)^2$$

and $N_{str} = dim\ v + dim\ \xi$ is the dimension of (v, ξ) space. We suppose the innovation rate $p_{inn} > 0$ is small and we use the linear relation $N_{reg}(t) = N_0 + p_{inn}t$, that gives

$$N_{reg}(t_0)^\gamma \geq N_0^\gamma(1 + p_{inn}\gamma t_0)).$$

Let $N_0 >> 1$ be the initial size of the gene regulation system. We replace the factor $\sigma_v(t_0)$ on its value at the initial moment and then the series (A27) reduces to a geometric progression. We find the sum of the series (A27) by the last relations, (A33), and formula (A38). Using the assumption that the random stress perturbations $\xi(t)$ are independent for different t, one has

$$\log P_{v,T} = \sum_{t_0=0}^{T} \log(1 - P_{out}(N(t_0))) \approx -\sum_{t_0=0}^{T} P_{out}(N(t_0)),$$

where $P_{out}(N)$ is the probability of leaving the homeostasis domain for the system with the regulation network of the size N. Here we use the asymptotics $\log(1 - P_{out}) \approx -P_{out}$ because P_{out} is small for large N. By (A38) and the above estimate for N_{reg}^γ the last estimate gives

$$\log P_{v,T} < -\sum_{t_0=0}^{T} \exp\big(-C_0 N_0^\gamma(1 + \gamma p_{inn} t_0)\big).$$

By computing the geometric series in the right-hand part, we obtain finally

$$\log P_{v,+\infty} \approx -\exp(-C_0 N_0^\gamma)\big(1 - \exp(-C_0\gamma p_{inn}^{-1} N_0^\gamma)\big)^{-1}. \tag{A39}$$

We see thus the viability (survival) probability depends sharply on the innovation rate p_{inn} and on the size of the network of gene regulation.

0.4.1 Toy example

To illustrate the general approach developed above, and find a connection between hard combinatorial problems with many constraints and biochemical kinetics, we consider a toy example using the classical model of the Michaelis-Menten reaction proposed by J. B. S. Haldane and G. E. Briggs. Let us consider the following equation:

$$\frac{d[ES]}{dt} = k_1[E_0][S] - (k_{-1}[S] + k_2)[ES],$$

where

1. $[S]$ denotes the concentration of a substrate;
2. $[ES]$ denotes the concentration of a enzyme-substrate complex;
3. $[E_0]$ denotes the constant, which is equal tos$[E] + [ES]$;

4. k_1 is the rate constant for the reaction of formation of an enzyme-substrate complex from an enzyme and a substrate;

5. k_{-1} is the rate constant for the dissociation reaction of an enzyme-substrate complex into an enzyme and a substrate;

6. k_2 is the rate constant for the reaction of converting an enzyme-substrate complex into an enzyme and a product.

In order to simplify the notation, we rewrite it to the form:

$$\frac{dv}{dt} = a\xi - (b_0\xi + b_1)v, \tag{A40}$$

where $\xi = [S], v = [ES]$ and a, b_0, b_1 are positive parameters, which can depend on temperature and other external parameters and also depend on a gene expression string s. Here our aim is to investigate viability with respect to variations of substrate ξ variations. We suppose that $\xi = \bar{\xi} + \sigma_\xi d\tilde{\xi}(t)/dt$, where $\tilde{\xi}(t)$ is the standard Wiener process and σ_ξ is a parameter of the noise magnitude. The equilibrium solution has the Michaelis-Menten form $\bar{v} = \frac{a\bar{\xi}}{b_0\bar{\xi}+b_1}$. We take the homeostasis domain D_H as an interval v_{min}, v_{max}. We are seeking v in the form $\bar{v} + \tilde{v}(t)$ supposing that corrections $\tilde{v}$ are small. Then removing terms $\tilde{\xi}\tilde{v}$, we obtain the linear differential stochastic equation

$$d\tilde{v} = \alpha\tilde{v}dt + \beta_0 d\tilde{\xi}, \tag{A41}$$

where

$$\alpha = b_0\bar{\xi} + b_1, \quad \beta_0 = a - b_0\bar{v} = \frac{ab_1}{b_0\bar{\xi} + b_1}.$$

Eq. (A41) describes a scalar Ornstein-Ulenbeck process, which is a Gaussian one with zero mean, therefore, to describe their properties, it is sufficient to compute the standard deviation of $\tilde{v}(t)$, which is $\sqrt{E\tilde{v}(t)^2}$. For large t and $\tilde{v}(0) = 0$ we have

$$E\tilde{v}(t)^2 = \sigma_\xi^2 \int_0^t \int_0^t \exp(-\alpha(t - t_1)) \exp(-\alpha(t - t_2)) E\big[\frac{d\tilde{\xi}(t_1)}{dt_1} \frac{d\tilde{\xi}(t_2)}{dt_2}\big] dt_1 dt_2.$$

Since $\frac{d\tilde{\xi}}{dt}$ is a standard white noise one has

$$E\big[(\frac{d\tilde{\xi}(t_1)}{dt_1} \frac{d\tilde{\xi}(t_2)}{dt_2}\big] = \delta(t_1 - t_2)$$

and for large t we obtain

$$E\tilde{v}(t)^2 = \frac{\sigma_\xi^2 \beta_0^2}{2\alpha} = \frac{\sigma_\xi^2 a^2 b_1^2}{(b_0\tilde{\xi} + b_1)^3} = \sigma_v^2.$$

(this is a particular case of formula (A37)). Suppose $\bar{v} \in (v_{min}, v_{max})$ (otherwise the homeostasis is violated). Let $R_0(\bar{\xi}) = \min\{\bar{v}(\bar{\xi}) - v_{min}, v_{max} - \bar{v}(\bar{\xi})\}$. Then the quantity ΔW is

$$\Delta W(\bar{\xi}, s) \approx \frac{R_0(\bar{\xi})^2}{2\sigma_v^2}.$$

This quantity depends on the gene expression s via the coefficients a, b_0 and b_1. If the $\bar{\xi}$ is a random quantity with the probability density function $\rho(\bar{\xi})$, we find that

$$P_{out}(s) \approx \int_{\xi_{min}}^{\xi_{max}} \rho(\xi) \exp\big(-\Delta W(\bar{\xi}, s)\big) d\xi. \tag{A42}$$

where

$$\xi_{min} = \frac{b_1 v_{min}}{a - v_{min} b_0}, \quad \xi_{max} = \frac{b_1 v_{max}}{a - v_{max} b_0}.$$

The integral in (A42) can be approximated by a finite sum that allows us to obtain an analogue of relations (A27), (A29).

We can conclude with the following. Even in the case of the simplest Michaelis-Menten reaction going in one stage and with a single substrate, the dependence of the survival probability on the genotype is quite complex and therefore it is easier to model it using semi-phenomenological models considered in the next sections.

Whatever the genetic expression, in the case when one resource is used, its unlimited fluctuations lead sooner or later to the death of the organism. Therefore, there should be a tendency to use more and more new resources.

An increase in the number N_g of genes controlling the reaction leads to an increase in ΔW, that in turn, results in approaching viability probability $P_{v,T}$ to 1 (as a result of gene duplication for example).

In the next sections, we consider semi-phenomenological gene-trait maps.

0.5 Examples of gene-trait maps

0.5.1 Single layered perceptron (SLP)

This model is based on an analogy with neural networks [10]. Consider the gene-trait maps defined by

$$f_j = \sigma(\sum_{i=1}^{N} w_{ji} s_i - h_j), \tag{A43}$$

where $j = 1, \ldots, M$. Here $\sigma(z)$ is a sigmoidal function of z such that σ is monotone increasing, $\sigma(z) \to 1$ as $z \to +\infty$ and $\sigma(z) \to 0$ as $z \to -\infty$. The coefficients w_{ij} can have different signs. If $w_{ji} > 0$ then the i-th gene is an activator for j-th trait, if $w_{ji} < 0$ it is a repressor, and for $w_{ji} = 0$ that gene does not affect the i-th trait. This model can be considered as a circuit with the possibility to regulate phenotype robustness via special parameters h_j, which are thresholds first introduced [11]. The model (A43) and certain similar ones are considered in [12, 13, 14].

Suppose that all w_{ij} are independent, normally distributed, random quantities with zero mean and variance r^2. These assumptions (standard for such models [15]) mean that we consider a random gene regulation.

Note that the Boolean linear programming problem applied in the paper to describe symbiosis, can be formulated as a problem to find the maximum of the following fitness functions:

$$F_{int} = \exp(\gamma \sum_{i=1}^{M} \log \sigma(\sum_{l=1}^{n} a_{il} u_l - h_i) - \sum_{l=1}^{n} r_l u_l) \qquad (A44)$$

where $r_l > 0, a_{il}, h_i, \gamma > 0$ are coefficients, and we are seeking for $u_l \in \{0, 1\}$. This fitness is similar to the SLP model.

0.5.2 Multilayered perceptron (MLP)

Here we describe functions $f_j(s)$ that are general enough to approximate any conceivable gene-trait maps.

To extend the possibilities of the SLP (which fails to approximate all logical functions [16]) one can use the MLPs. MLPs have one or several hidden layers organized in a feed-forward cascade; genes in each layer depend recursively on the genes in the previous layer via sigmoidal functions. For instance, during *Drosophila melanogaster* morphogenesis several transcription factors act in a cascade: maternal factors activate gap or pair-rule genes; gap and pair-rule genes activate Hox genes; Hox genes activate realisator genes that cause the segments in the developing embryo to differentiate. The MLP architectures thus mimic realistic gene regulatory networks, which we encounter in developmental biology. Furthermore, the MLP architectures are Turing-complete: they can approximate any Boolean function, hence any conceivable gene-trait map [17].

A general d-layered perceptron model can be defined by the iterative relations

$$f_j^{(l)}(s) = \sigma(S_j^{(l)} - h_j^{(l)}), \quad S_j^{(l)} = \sum_{i=1}^{N_l} w_{ji}^{(l)} f_i^{(l-1)}, \qquad (A45)$$

where $l = 1, \dots, d$,

$$f_j^{(0)}(s) = s_i, \qquad (A46)$$

where l are levels of the control. For the top-level of control, $l = d$, $f_j^{(d)} = f_j$ are final phenotypical traits involved in the fitness defined by Eq. (A12), whereas at the bottom level $f_i^{(0)} = s_i$ are the coding genes.

To understand how the MLP can serve as a model of metabolism, let's consider two metabolic pathways working cooperatively. Let each pathway be represented as a chain of Michaelis-Menten reactions, defined by (A4). The outputs of both chains are v_1^{out} and v_2^{out} and the dependencies of the v_i^{out} productions on the genotype s are given by sigmoidal functions (A43). Both products serve as inputs for a two-substrate Michaelis-Menten reaction (A5), producing the final

output of the entire metabolic system. As a result, for P_v we obtain a model resembling a MLP.

In the next subsection, we demonstrate that both single and multilayered perceptron models can replicate the topological characteristics of rugged, fragmented fitness landscapes observed in reality. To this end, we consider the widely recognized complex combinatorial model (K-SAT), which can be derived from the single-layered perceptron.

0.5.3 K-SAT

K-SAT (K-satisfiability problem) is a basic hard combinatorial problem [18] having a transparent biological interpretation. Biologically, it describes M constraints, each of which depends on K Boolean genes and must be satisfied by the correct choice of the K genes involved. The number of satisfied constraints can be considered as the fitness of a Boolean string of length $N >> K$.

Remind a formulation of K-SAT in more detail. Let K be a positive integer, and each Boolean trait f_i is determined by K genes $s_{i_1}, s_{i_2}, \ldots, s_{i_K}$, which can be switched on or off. Let us further suppose that to achieve $f_i = 1$, it is sufficient to correctly switch at least one of those genes. In mathematical terms, this implies that the Boolean function f_i is a disjunction of literals $\tilde{s}_{i_1}, \tilde{s}_{i_2} \ldots \tilde{s}_{i_K}$, where the literal $\tilde{s}$ is either s itself or its negation $\bar{s} = 1 - s$. The K-SAT problem involves finding a Boolean string (genotype) s such that all $f_i = 1$, where $i = 1, 2, ..., M$, where $i = 1, 2, ..., M$ (see [18] for more details). The parameter K is the number of genes involved in the regulation of a trait. In biology, K-SAT was considered first as a speciation model in [19]. Note the K-SAT can be obtained from SLP as a hard limit, where $\sigma(y) = 1$ for $y > 0$ and $\sigma(y) = 0$ for $y < 0$, and under a special choice of thresholds h_i [12]. Consider a SLP, where $w_{ij} \in \{0, 1, -1\}$, and for each i exactly K of $w_{ij} \neq 0$. We set $h_i = k_i - 1/2$, where k_i is the number of negative w_{ij}. Then f_i defined by (A43) is a disjunction of K literals.

The K-SAT problem is very well-studied beginning with the seminal work [20] (see [18], Ch. 14 for an overview). The topology of the fitness landscape for K-SAT of a random structure with a large M is completely determined by two parameters: K and $\alpha = M/N$. An increase of α leads to more and more rough fitness landscapes and, as a consequence, to many remarkable phenomena ([18], Ch. 14). There is a critical value α_c such that for $\alpha > \alpha_c$ this landscape is too rugged and, as a result, the solution s satisfying $f_i(s) = 1$ for all i does not exist.

Let $\alpha < \alpha_c$ and the well-adapted phenotype almost surely exists. How many evolution steps N_{evol} are needed to find that genotype? It is well known that K-SAT is an NP-complete problem for $K > 2$ [21, 22, 18], i.e., the running time to resolve it is, in general, too large (increases exponentially in the trait number M). Following the basic ideas of Valiant [23, 24], we assume evolution is feasible if the number N_{evol} of evolutionary steps needed for the fitness maximization increases as a polynomial of M, and non-feasible if that number is exponential in M. One can show that for the K-SAT model there is a phase transition at $\alpha_{alg} < \alpha_c$ such that if $\alpha > \alpha_{alg}$ then evolution is not feasible. Note that for

$\alpha \in (\alpha_{alg}, \alpha_c)$ a well-adapted organism exists but the evolution is not capable of finding the corresponding genotype within a reasonable time interval.

0.6 Models of cooperation

0.6.1 Models for a simple symbiosis

Let us consider organisms $O_1, ..., O_m$, which coexist in an external environment. They can activate various genotypes $s^{(1)}, s^{(2)}, ..., s^{(n)}$, with the help of which they produce chemical compounds (gene products) necessary for their survival. Suppose a_{il} is the quantity of a gene product x_i, which can be obtained by the genotype $s^{(l)}$, r_l is the cost of resources needed to activate the genotype $s^{(l)}$ and h_i is the minimal amount of i-th product needed for the organism survival. In mathematical terms, the organism cooperation problem reduces then to the following Integer Linear Programming problem: *To minimize*

$$F_{targ}(u) = \sum_{l=1}^{n} r_l u_l \tag{A47}$$

under conditions

$$\sum_{l=1}^{n} a_{il} u_l \geq h_i, \quad \forall i = 1, ..., M \tag{A48}$$

$$u_l \in \{0, 1\}. \tag{A49}$$

This means that each organism produces a subset of gene products and all together they should produce all those products needed for survival using the minimal resource amount $F_{targ}(u)$. The Boolean unknown $u_l = 1$ if the genotype $s^{(l)}$ is activated otherwise $u_l = 0$. Note that we can consider another variant of the problem, where the relation (A47) is replaced by

$$F_{targ}(u) < C_{res}. \tag{A50}$$

This means that the population of organisms survives with a restricted amount of resources. In the particular case $a_{il} \in \{0, 1\}$, $r_l = 1$ and $h_i = 1$, the Integer Linear Programming problem reduces to the Set Cover Problem [25]. Consider the Set Cover Problem and its biological interpretation in more detail.

Let ξ be a parameter, which describes the state of the environment. We suppose that there is a set $\mathcal{U} = \{f_1, ..., f_M\}$ of all possible ecological constraints (Universe) and for different ξ there occur different subsets of constraints $\mathcal{E}(\xi) \subset \mathcal{U}$.

Let us denote by $\mathcal{F}$ a family consisting of n subsets $\mathcal{E}_k$, $k = 1, ..., n$ of the Universe $\mathcal{U}$. Suppose k-th organism is capable of satisfying constraints from a set $\mathcal{E}_k \in \mathcal{F}$ belonging to that family. We suppose that

$$\mathcal{U} = \bigcup_{k=1}^{n} \mathcal{E}_k, \tag{A51}$$

i.e., all the sets $\mathcal{E}_k$ cover the whole universe $\mathcal{U}$.

Consider different subfamilies $\mathcal{E}_j \subset \mathcal{F}, j \in J$, where J is a subset of $[1, n] = \{1, ..., n\}$. Let us denote by $|J|$ the number of elements of the set J, i.e., the size of the subfamily. Then the Set Covering Problem is as follows:

Consider the family $\mathcal{F}$ of subsets of $\mathcal{U}$. Suppose (A51) is satisfied. For a subset $\mathcal{E} \subset \mathcal{U}$ to find a cover $\mathcal{E}_j$ of $\mathcal{E}$ by subsets $\mathcal{E}_j \in J$:

$$\mathcal{E} \subset \bigcup_{j \in J} \mathcal{E}_j, \tag{A52}$$

that has a minimal size $|J|$.

In other words, given a set $\mathcal{U}$ of elements $\{1, 2, ..., M\}$ (the Universe) and a collection $\mathcal{F}$ of m sets whose union equals the Universe, the problem is to find the smallest sub-collection of $\mathcal{F}$ whose union equals the Universe (this collection may not be the only one).

Note that both Integer Linear Programming and Set Covering Problems are NP-hard [26, 27], nonetheless, there exist different methods to find approximating solutions to Integer Linear Programming and Set Covering Problems [27]. For example, one can use approximate solutions of the Integer Linear Programming problem by solutions of an appropriate linear programming problem that can be effectively resolved in polynomial time, or one can apply greedy and genetic algorithms.

0.6.2 Relaxation of Integer Linear Programming

One of the effective methods to resolve an Integer Linear Programming Problem is a relaxation approach. We replace restrictions (A49) with weaker ones $u_l \in [0, 1]$, where u_l are real-valued unknowns. Then we obtain the following Relaxed Linear Programming problem: *To minimize wasted resources $F_{targ}(u)$ under restrictions*

$$\sum_{l=1}^{n} a_{il} u_l \geq h_i, \quad \forall i = 1, ..., M \tag{A53}$$

$$0 \leq u_l \leq 1. \tag{A54}$$

0.6.3 Genetic algorithms

Genetic algorithms (GA's) are inspired by the process of natural selection and to perform genetic algorithms for ILP and SCP, we need fitness. The following relation can define a natural fitness function associated with our Integer Linear Programming:

$$F(s) = g(\sum_{l=1}^{n} r_l u_l) \prod_{i=1}^{M} H\Big(\sum_{l=1}^{n} a_{il} u_l - h_i\Big), \tag{A55}$$

where $H(z)$ stands for the step function, which equals 1 if $z > 0$ and 0 otherwise, and $g(R)$ is a decreasing smooth function of consumed resources $R = \sum_{l=1}^{n} r_l u_l$. The product of factors H means that our system does not survive when conditions (A53) are violated. The factor g expresses the property that the fitness

of colonies consuming fewer resources is larger. Note that the function $\log F(s)$ can be approximated by the SLP model. In fact, let us approximate $H(z)$ by a soft step function $\sigma_a(z) = (1 + \exp(-az))^{-1}$ with a large a. It can be considered as a soft variant for constraints. For the factor g we use the same idea: $g(R) = \sigma_b(R)$. Then

$$\log F(s) \approx \sum_{i=1}^{M} f_i(\sum_{l=1}^{n} a_{il}u_l - h_i) + \tilde{f}(\sum_{l=1}^{n} r_l u_l),$$

where $f_i, \tilde{f}$ are piece-wise linear functions (similar to ReLu sigmoid).

Our GA uses the following techniques [28, 29]:

1. Single point crossover is a crossover point on the parent organism string is selected. All data beyond that point in the organism string is swapped between the two parent organisms;

2. Tournament selection is the selection that involves running several "tournaments" among a few individuals chosen at random from the population. The winner of each tournament (the one with the best fitness) is selected for crossover.

3. Bit string mutation: we use mutations of bit strings going through bit flips at random positions.

0.6.4 Greedy algorithm for symbiosis

There is a greedy algorithm for the polynomial-time approximation of Set Covering that chooses sets according to the following natural rule: at each stage, let us choose the set that contains the largest number of uncovered elements [25]. This method can be implemented in time linear in the sum of the sizes of the input sets. Let $Harm(d) = \sum_{i=1}^{d} 1/i$ be the sum of the first d terms of harmonic series.

Then the greedy algorithm achieves an approximation ratio of $Harm(d_*)$, where $d_* = \max |\mathcal{E}_k| \in \mathcal{F}$. The approximation ratio measures the extent to which the value of the target function achieved by the approximate solution compares to that of the optimal solution.

0.6.5 Jacob–Monod: Biological realization of greedy algorithm

The greedy approach can be interpreted as follows: each organism tries to satisfy constraints that are still not satisfied by others that allow maximizing the organism's individual fitness.

Such behavior can be performed by the gene regulation first described by J. L. Monod and M. Jacob [30]. In the Jacob–Monod model, the *Escherichia coli* lac operon encodes proteins necessary for the transport and breakdown of the sugar lactose (lac). The production of proteins is prevented when a repressor, encoded by a regulatory gene, binds to its operator, a specific site in the DNA

sequence that is close to the genes encoding the proteins. So, if each organism has a sensor permitting to find which products are already produced by other organisms and therefore there is no necessity to produce them then to maximize the fitness, the organism must produce the maximum number of products not created by other organisms.

0.6.6 Biological realization of the relaxation

In this subsection, we show that the Linear Programming problem defined by conditions (A53), (A54) and the target function (A47) can be associated with a biological model, which describes a primitive multicellular organism functioning.

Let us consider an organism consisting of cells located at positions $x \in \Omega \subset \mathbb{Z}^3$, where Ω is a bounded set. Let $u_l(x) = 1$ if a cell of l-th type occupies the position x. Cells produce the reagents u_i so that the general amount of i-th reagent is

$$\bar{u}_i = \sum_{l=1}^{n} \sum_{x \in \Omega} a_{il} u_l(x). \tag{A56}$$

We suppose that each position can be occupied by a single cell only:

$$u_l(x) \in \{0, 1\}, \quad u_j(x) = 1 \implies u_l(x) = 0 \quad \textit{for } l \neq j. \tag{A57}$$

Let us denote by $\langle u_l \rangle$ the averaged number of cells of l-th type:

$$\langle u_l \rangle = |\Omega|^{-1} \sum_{x \in \Omega} u_l(x),$$

where $|\Omega|$ the number of points x in Ω. Then a natural viability condition $\bar{u}_i > \bar{h}_i$ can be rewritten as follows:

$$\sum_{l=1}^{n} a_{il} \langle u_l \rangle > h_i, \tag{A58}$$

where $h_i = \bar{h}_i / |\Omega|$. If our organism is large, i.e. $|\Omega| >> 1$ then one can consider $\tilde{u}_l = \langle u_l \rangle$ as real valued variables. Thus, we obtain the following LP for $\tilde{u}_l \in \mathbb{R}$:

Under restrictions

$$\sum_{l=1}^{n} a_{il} \langle u_l \rangle > h_i, \quad \forall i = 1, ..., M \tag{A59}$$

$$0 \leq \langle u_l \rangle \leq 1 \tag{A60}$$

to minimize $F_{targ}(\langle u_l \rangle)$.

The remarkable fact is that, in contrast to Integer Linear Programming, this problem is not NP-hard. If this problem has a solution, then for large $|\Omega|$ we can find an organism corresponding to this solution.

0.7 Complexity barriers

In this section, we first consider the evolution complexity barriers for different models without cooperation. Furthermore, we consider cooperative effects and the following transitions: from simplest unicellular organisms (prokaryotes) to symbiotic cooperation of those organisms, further to colonies of bacteria with a simple genetic regulation and colonies of cells with a more sophisticated genetic regulation.

0.7.1 Upper bound for complexity in the case A

In this subsection, we consider the following problem: whether there is a well-adapted phenotype or not for organisms in individuality (without cooperation). Let us formulate the main assumption.

Assumption RG (on the randomness of genetic regulation) *Random functions $f_i(s, \mathbf{w}, \xi)$ satisfy*

$$E[f_i(s, \mathbf{w}) f_j(s, \mathbf{w})] = E[f_i(s, \mathbf{w})] E[f_j(s, \mathbf{w})], \quad \forall i \neq j, \tag{A61}$$

where $E[f_i]$ denotes the expected values of f_i taken with respect to the density $\mu(\mathbf{w})$.

Here μ denotes the probabilistic measure that defines the random distribution of the parameters $\mathbf{w}$ of genetic regulation network. As an example, we can consider such a distribution for parameters w_{ji} of the SLP model, where $j = 1, ..., M$ and $i = 1, ..., N$. We take $|w_{ji}| = 1$ with the probability K/N, where $N >> 1$ and the number $K > 0$ (no obligatory integer) has the order 1 (the averaged number of genes controlling a given trait). If $|w_{ji}| = 1$, we, by a fair coin, choose either $w_{ij} = 1$ or $w_{ij} = -1$. We can set $h_j = 0$ or $h_j = \bar{h}$, or take random h_i, distributed for example by normal law. It is easy to check that then Assumption **RG** holds.

This assumption can be interpreted as the randomness of gene regulation. Let us define the quantity $P_{+,\delta}$ by

$$P_{+,\delta} = \max_{i \in [1,M], \ s \in S^N} \Pr[f_i(s, \mathbf{w}, \xi) > 1 - \delta], \tag{A62}$$

where $\delta \in [0, 1)$ is a small parameter, $[1, M] = \{1, 2, ..., M\}$ and $S^N = \{0, 1\}^N$ the set of all genotypes.

If $P_{+,\delta}$ is not small there exists a genotype s satisfying conditions $f_i(s) > 1 - \delta$ for all $i = 1, ..., M$, i.e., sufficiently well adapted with respect to all M constraints.

Under Assumption **RG** one can obtain the following upper bound for M:

$$M < C_{\max} N, \quad C_{\max} = \frac{\ln 2}{|\ln P_{+,\delta}|}, \tag{A63}$$

where N is the number of coding genes and a constant $C_{\max}$ is uniform in M, N as $M, N >> 1$. If condition (A63) does not hold, the well-adapted phenotype is

absent, with a probability exponentially close to 1 (see subsection 0.7.2, proposition I). Therefore, inequality (A63) establishes the upper limit of the organism's complexity, which can be achieved without cooperation between individual cells. The maximal number of the traits (constraints to be adapted) does not exceed the number of genes multiplied by $C_{\max}$. Our result implies that if M ecological constraints are given, to satisfy all of them one can only if $M < C_{\max}N$, even when evolution goes a very long time.

In the next subsections, we formulate the rigorous result and state the proof of estimate (A63). Furthermore, we compute the concrete values of $C_{\max}$ for K-SAT, SLP and MLP models. Note that for MLP the coefficient $C_{\max}$ may be much bigger than for single-layer models, i.e., cascade regulation may diminish the complexity cost.

0.7.2 General bounds for maximal complexity

We are seeking for a δ-optimal genotype $s_{opt,\delta}$ such that

$$f_j(s_{opt,\delta}) > 1 - \delta, \quad \forall j = 1, ..., M. \tag{A64}$$

where $\delta > 0$ is small.

Similarly to the K-SAT model, we use the parameter $\alpha = M/N$.

There holds

Proposition I. *Let assumption* **RG** *hold. Then if*

$$\alpha = M/N > C_{\max} := \frac{\ln 2}{|\ln P_{+,\delta}|} \tag{A65}$$

the probability Pr_{sat} to satisfy (A64) is exponentially small

$$Pr_{sat} < \exp(-c_0 N), \tag{A66}$$

where $c_0 > 0$ is a constant uniform in N.

Let us compare our relation (A65) with known results for K-SAT. For K-SAT model we obtain that $Prob\{f_i(s) = 1\} = 1 - 2^{-K}$ for all s. Therefore for large K

$$C_{\max} < \frac{\ln 2}{|\ln(1 - 2^{-K})|} \approx 2^K \ln 2,$$

that coincides with the well known results (see [18], Ch. 14, page 750).

0.7.3 Proof of Prop. I

We use arguments analogous to K-SAT theory [20, 18]. To derive a upper estimate of $C_{\max}$, we apply the Markov inequality (A67): for each positive random quantity Y and each $a > 0$

$$\Pr\{Y > a\} \leq \frac{E[Y]}{a}. \tag{A67}$$

Let $X(s)$ be a random quantity defined by: $X(s) = 1$ if $f_i(s, \mathbf{w}) > 1 - \delta$ for all i and $X(s) = 0$ otherwise. Let us set $Y = \sum_{s \in S^N} X(s)$. Note that the Y takes non-negative integer values and it is greater than 0 if and only if there is a solution s satisfying (A64). Moreover,

$$X(s) = \prod_{i=1}^{M} H(f_i(s, \mathbf{w}) - 1 + \delta),$$

where $H(y) = 1$ for $y > 0$ and $H(y) = 0$ otherwise. Then by inequality (A67) one obtains

$$Prob\{Y \geq 1\} \leq E[Y] = \sum_{s \in S^N} E[\prod_{i=1}^{M} H(f_i(s, \mathbf{w}) - 1 + \delta)]. \tag{A68}$$

Note that due to (A61)

$$E\left[\prod_{i=1}^{M} f_i(s)\right] = \prod_{i=1}^{M} E[H(f_i(s, \mathbf{w}) - 1 + \delta)],$$

By definition (A62) we find that

$$Prob\{Y \geq 1\} \leq 2^N \exp\left(M \ln P_{+,\delta}\right). \tag{A69}$$

Note that

$$\ln\left(2^N \exp\left(M \ln P_{+,\delta}\right)\right) = N \ln 2 + M \ln P_{+,\delta}. \tag{A70}$$

Let $N >> 1$ and $M >> 1$ but $\alpha = M/N = O(1)$. Under those conditions let $C_{\max}$ be the maximal value of α such that the probability to satisfy all constraints $f_i(s) = 1$ is not exponentially small.

Then, repeating standard arguments (see [18], Ch.14) we find by (A69) and (A70) that $C_{\max}$ satisfies the inequality (A65). This completes the proof. Further, we compute the complexity bound $C_{\max}$ for different models.

0.8 Complexity barrier for different models

0.8.1 Complexity barrier for SLP models

Here we take a very small δ and omit dependencies on this parameter it in notation. Let us apply Prop. I to derive estimates of the complexity bound $C_{\max}$ for the shallow perceptron model with a single layer. Moreover, we assume for simplicity that $h_i = h$ for all i. According to Eq. (A65) we compute P_+. To this end, let us consider the quantities

$$\tilde{P}_{+,j}(s) = Prob\{y_j(s) > h\}, \tag{A71}$$

where y_j are defined by $z_j = \sum_{i=1}^{N} w_{ji} s_i$. We note that the random quantities y_j have normal densities, with $E[z_j] = 0$ and $Var\ z_j(s) = Nr^2$ and $\tilde{P}_{+,j}(s)$ does

not depend on j and s. Therefore, the quantity P_+, which is the maximum of all $\tilde{P}_{+,j}(s)$ over all j, s is given by

$$P_+ = 2\pi^{-1/2} \int_{\bar{h}}^{\infty} \exp(-u^2/2) du, \tag{A72}$$

where $\bar{h} = h(\sqrt{N}r)^{-1}$. The quantity $\bar{h}$ can be called normalized, or effective, threshold, and it defines the trait f_i sensitivity with respect to mutations.

For K-SAT our assertion gives the following relation :

$$C_{\max} \approx 2^K \ln 2, \quad K >> 1. \tag{A73}$$

It is consistent with the well-known results (see [31, 18]).

0.8.2 Complexity barrier for layered models

To simplify the statement we consider the case $d = 2$. The most interesting situation arises for negative threshold parameters $h_i^{(l)} = h^{(l)}$, where $l = 1, 2$ such that $|h^{(l)}| >> \sqrt{N}r$. We suppose that all $w_{ij}^{(l)}$ are independent, normally distributed, random quantities with zero mean and variance r^2, and $h_i = h$, $h < 0$ and $|h| >> \sqrt{N}r$.

Let us introduce the probabilities:

$$P_+^{(1)}(v) = Prob\{W_{1,j}(v) > h^{(1)}\}, \quad P_+^{(2)}(s) = Prob\{W_{2,j}(s) > h^{(2)}\}. \tag{A74}$$

where

$$W_{1,j} = \sum_{i=1}^{N_1} w_{ji}^{(1)} v_i,$$

$$v_i = H(W_{2,i}(s) - h^{(2)}), \quad W_2(s) = \sum_{l=1}^{N} w_{il}^{(2)} s_l.$$

We observe that under our assumptions to $h^{(1)}$ the probability $P_+^{(2)}(s)$ is close to 1. Consequently, $v_j \approx 1$ for each j and the random quantity $W_{1,j}(v)$ have normal densities $\mathbf{N}(0, a^2)$ with $a = r\sqrt{N}$. Therefore, repeating computations of subsection 0.8.1 we find

$$C_{\max} \approx \sqrt{2\pi} \ln 2 \; \bar{h}_1 \exp(\bar{h}_1^2), \quad \bar{h}_1 = h^{(1)}/\sqrt{N}r. \tag{A75}$$

Assuming for simplicity that $h^{(2)} = h$, $h^{(1)} = h$ let us compare the values of $C_{\max}$ for the MLP with such thresholds and for the SLP with the same values r, h. Comparing the last relation we observe that in the case MLP the complexity bound is proportional to $\exp(\bar{h}^2)$ while for SLP that bound is proportional to $exp(\bar{h}^2)/2$. Thus we conclude that for the MLP the complexity bound is essentially higher with respect to the case of SLP with the parameters. Just by inserting a new layer, one can increase the complexity barrier $C_{\max}$ and this effect is exponential. This means that the cascade regulation may be more effective.

0.9 Some estimates of complexity barriers for cooperative models

0.9.1 Case B

Let us consider first the case **B**. We consider a symbiosis by the Set Covering Model with a Universe $\mathcal{U}$. This means that we have the set $\mathcal{U}$ of environmental constraints for the whole group of organisms. Each organism can satisfy a subset $\mathcal{E}_k \subset \mathcal{U}$ consisting of M/n constraints only. Then by (A63) we obtain that the gene number N satisfies the estimate

$$N > \frac{\beta M}{n} + N_{reg}, \quad \beta = C_{\max}^{-1}, \tag{A76}$$

where N_{reg} the number of genes s^{reg} needed to support cooperation (symbiosis). For example, for a Major Evolutionary Transition (MET) (on MET's see [32, 33]) leading from prokaryotes to eukaryotes by symbiosis, the genes s^{reg} provide adhesion of different organelles (for example, mitochondrion). As it is shown above, without cooperation we have

$$N > \beta M. \tag{A77}$$

So, the transition from **A** to **B** is computationally profitable if

$$N_{reg} < \beta \frac{(n-1)M}{n}. \tag{A78}$$

One can expect that this inequality holds for hard environments with $M >> 1$ (see below an analysis for Jacob–Monod regulation). Further analysis leads to intriguing conclusions. Suppose that we have a gene redundancy: each feature is controlled by K genes. In this case, we can use K-SAT model. Then the parameter $\beta \approx 2^K \ \ln 2$. Let $n = 2$ (for example, we simulate symbiosis between a prokaryote and mitochondrion). This means that if $N_{reg} < 2^K M/2$ then the transition $\mathbf{A} \to \mathbf{B}$ is profitable. We conclude thus that gene redundancy (which can appear as a result of gene duplications) sharply facilitates symbiosis.

0.9.2 Case C

Consider conditions (A48) and (A49). Suppose they can be satisfied by a Boolean choice $u = \bar{u}$. Each u_l is regulated by a gene s: $u_l = u_l(s)$. The relation $u_l(s) = 1$ means that l-th cell produces the gene products belonging a set $\mathcal{C}_l$. Under assumption **RG** we obtain then (see the end of subsection 0.7.1) that

$$N > M/n + N_{reg}. \tag{A79}$$

Let us estimate N_{reg} for the Jacob–Monod regulation. Note that to perform this regulation each cell should have sensors that are capable of recognizing M/n different reagents. So, one can expect that in this case N_{reg} has the order M/n. So, for minimal needed gene number N we obtain

$$N > C_{JM} \frac{M}{n} = N_{JM}, \tag{A80}$$

where C_{JM} is a constant > 1 while for individual cells we have $N = M >> N_{JM}$. So, the transition from **B** to **C** is computationally profitable if $n > C_{JM}$.

General arguments [34, 35] show that during evolution the number N of genes should have a tendency to increase and metabolic networks should increase. Indeed, for a network of a fixed structure the probability $P_v < 1 - \delta_0$, where δ_0 is a fixed positive number (possibly small). This means that this metabolic network functioning earlier or later will be violated. This simple but basic fact was first pointed out by M. Gromov and A. Carbone in [2]. They noticed that environment fluctuations inevitably destroy cells, but cell populations can be stabilized within very long time periods (maybe eternally) via cell replications. One can show formally that the gene number N should grow to provide this stability and metabolic networks are more stable if they grow to evolve toward the scale-free structure [36, 34, 35]. There exist a number of different theories on the mechanism of metabolic network growth (see review [32]). Let us note that if during evolution the gene number $N(t)$ has a tendency to increase (possibly, in a non-monotone way) then the constraint number M also grows. Indeed, to support a larger genotype, we need a larger metabolic network. This property of the network growth and the free-scale structure of networks leads to interesting effects, which can be interpreted as critical phenomena in growing networks.

Further, we consider the causes of transitions $\mathbf{A} \to \mathbf{B}$, $\mathbf{B} \to \mathbf{C}$ and $\mathbf{C} \to \mathbf{D}$ and possible biological mechanisms for their realization.

0.10 Transitions to cooperation forms

0.10.1 Transition to a simple symbiosis (A to B)

Consider, for example, a primitive biosphere consisting of prokaryote cell colonies. Those colonies survive under ecological constraints, which form the set of constraints $\mathcal{U}_M = \{\mathcal{E}_1, ..., \mathcal{E}_M\}$ and the cell genotype size N satisfies the condition $N > \beta M$, i.e., the environment hardness is beneath of the complexity barrier. Then those cells can be adapted to $\mathcal{U}_M$. Let an ecological catastrophe happen, and new conditions are defined by a new set $\mathcal{U}_{\bar{M}}$ with $\bar{M} > M$ such that $\mathcal{U}_M \subset \mathcal{U}_{\bar{M}}$ (this inclusion means that the new environment is harder).

To analyze the adaptation problem, let us introduce useful parameters. Let us denote by τ a characteristic time needed to invent and fix in populations a new gene useful for adaptation and by T a characteristic time of ecological changes. Then during those changes, evolution can invent at most $N_{new} \approx CT/\tau$ of new genes, where $C > 1$. Furthermore, there arise two cases: **S** slow environment changes and **F** fast ones.

According to (A77) without MET a gradual evolution should invent at least $N_{grad} = \beta\bar{M} - N$ genes to provide adaptation. With MET it suffices to invent $N_{coop} = \frac{\beta M}{n} + N_{reg} - N$ genes, where $n \geq 2$ is the number of types of cells involving in the symbiosis and N_{reg} the number of genes needed to support symbiosis. Note that N_{reg} does not depend on the environment, this number depends on cell properties: the corresponding genes support cell communications.

One can expect thus that for hard environments where $\bar{M} >> M >> 1$ we have $N_{MET} << N_{reg}$. Then fast environmental changes can be characterized by the condition

$$N_{new} < N_{grad} = \beta \bar{M} - N. \tag{A81}$$

In this case, gradual evolution without cooperation invention does not have time to create enough genes. If

$$N_{coop} < N_{new} < N_{grad}, \tag{A82}$$

then the transition **A** $\rightarrow$ **B** is more probable than a gradual evolution without transition. Our final conclusion is

The existence of an exponentially large complexity cost implies that organisms facing rapid environmental changes are confronted with a choice: either to develop regulatory genes or face extinction. The chance of survival through gradual evolution is exponentially small.

0.10.2 Transition to colonies with Jacob–Monod regulation (B to C)

Transitions from the simplest symbiosis system to cell colonies can occur as an answer to a resource decrease. To explain this mechanism mathematically, let us consider relations (A50) and (A83). If the constant C_{res} decreases then the Integer Linear Programming Problem may have no solutions u at all while LP can have solutions. Therefore, colonies of cells with the Jacob–Monod regulation are more adaptable to the environment than ensembles of non-interacting cells because they consume fewer resources.

0.11 Transition to a more sophisticated regulation (C to D)

The lack of resources for consumption may also explain the transition from cell colonies to more complicated multicellular organisms with gene regulation. We use the fact that Jacob–Monod regulation can be interpreted as a greedy algorithm and that the greedy algorithm, in general, is not optimal and does not permit finding an optimal value of the target function. Our target function is the number of consumed resources. As above, we again use relation (A50). If the critical constant C_{res} (which defines the maximal level of resource amount) falls beneath the value, which can be obtained by the greedy algorithm, then the simple cell colony does not survive. So, starving can be considered as a reason for a transition from simple cell colonies with Jacob–Monod regulation to multicellular organisms, where more sophisticated algorithms of gene regulation work.

0.12 Numerical simulations

Numerical simulations are consistent with our analytical considerations, but they also lead to some interesting conclusions. Firstly, let us note that for the

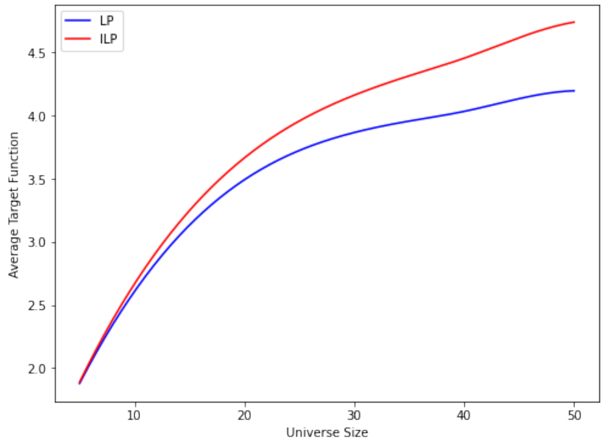

Figure A1: This figure shows that, in general, the minimal possible value of the target function for the linear programming problem is less than that value for the corresponding integer Linear programming problem (with the same coefficients).

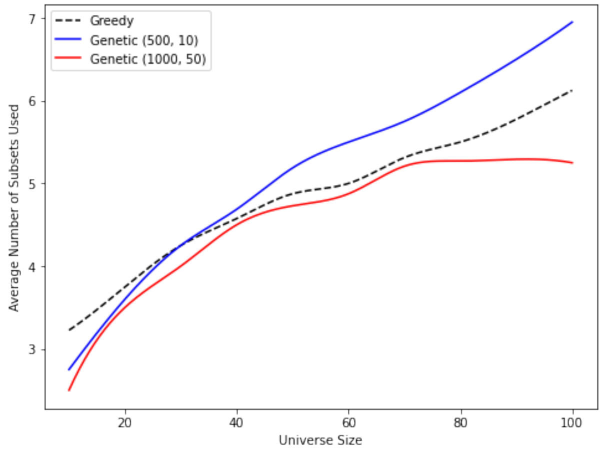

Figure A2: This plot shows how genetic and greedy algorithms use resources. Mathematically, resources are represented by the number N_{sub} of subsets required to cover a given set. We compare the performance of a greedy algorithm with various simulations of a genetic algorithm, which are defined by two parameters *the numbers* $(1000, 50)$ *indicate* 1000 *steps with populations of* 50 *members each*). To achieve optimal coverage, the most sophisticated genetic algorithm utilizes the smallest possible value of N_{sub}.

linear programming problem, the minimum of the target function $F_{targ}(u)$ is less than the corresponding minimum for integer linear programming with the same data:

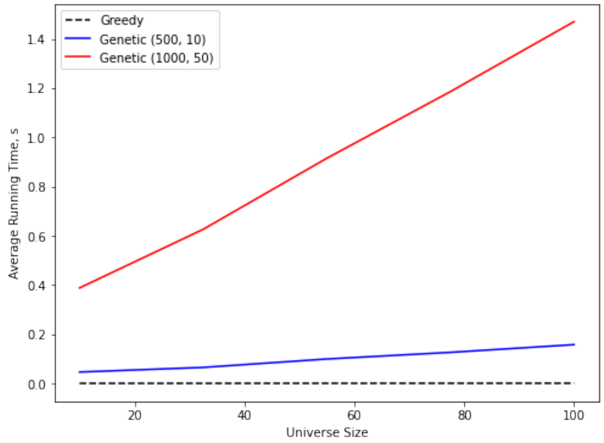

Figure A3: This plot displays the running times for the same genetic and greedy algorithms as in the previous Figure A2. We compare the number of elementary steps required to achieve the optimal value of the target function for the greedy algorithm with those of different simulations of the genetic algorithm. Both Figure A2 and this figure demonstrate that the greedy algorithm is faster than the genetic ones, but it utilizes resources less efficiently.

$$\min_{u:u_i\in[0,1]} F_{targ}(u) \leq \min_{u:u_i\in\{0,1\}} F_{targ}(u), \tag{A83}$$

where the left and the right minima are taken over all u satisfying the restrictions (A59) and (A60) and (A53), (A54), respectively. This fact is illustrated by Fig. A1.

Furthermore, it is important to note that, in general, solutions of the Set Covering Problem obtained by the greedy algorithm are not optimal.

Results are shown in Fig. A1–A5. The plots in Fig. A1 show that, in general, the minimal possible value of the target function for the Boolean programming problem is less than that value for the corresponding integer linear programming problem (with the same coefficients). This result can be translated into the biological language as follows. By our biological interpretations, we obtain that, to survive, the colony of cells with a simple cooperative behavior uses fewer resources (per cell per unit time) than cells that do not cooperate.

Results shown in Fig. A2 can be interpreted biologically as follows. A simple colony with a greedy algorithm of cooperation (that corresponds to Jacob–Monod regulation) consumes more resources than a more cunningly organized colony, where gene regulation is found as a result of a long adaptation process (mathematically, it is a genetic algorithm for adaptation).

However, being organized does not guarantee better results, as can be seen in this figure (blue line corresponds to a genetic algorithm that performs worse than greedy.

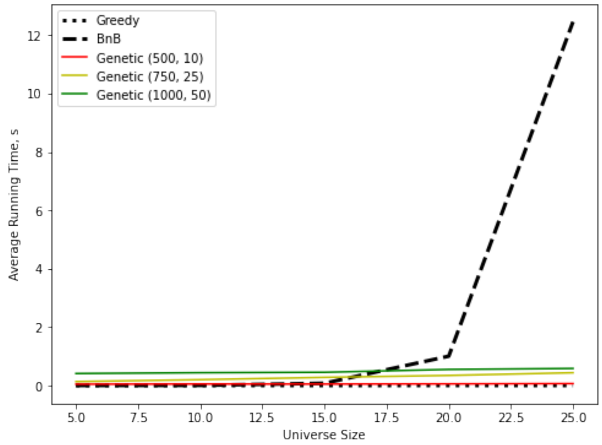

Figure A4: On this plot we see that the running time to find an optimal solution sharply increases as the Universe in Set Cover problem becomes bigger (too many ecological constraints). Therefore, evolution must use heuristic algorithms of adaptation such as gene regulation á la Jacob–Monod (mathematically it is a greedy algorithm). However, these algorithms sometimes fail to find the optimal solution. Therefore, one can expect that (particularly under rapid environment variations) organisms are not perfectly adapted, and not all properties of organisms can be explained by adaptation conditions.

On the other hand, Fig. A3 shows that adaptation for genetic algorithms takes a longer number of steps than for primitive greedy algorithms.

Further, we compare the performance of genetic and greedy algorithms with results obtained by the well-known Branch and Bound algorithm, which is popular for discrete combinatorial optimization problems. This algorithm is capable of finding optimal solutions (a feat that genetic and greedy algorithms generally fail to achieve), but it can degenerate into an exhaustive search for problems of large size, leading to excessively long running times. This characteristic is illustrated in Fig. A4. We can conclude that evolution functions heuristically, working as a tinkerer, as described in the famous essay by F. Jacob.

Fig. A5 displays the relative performance of different algorithms. Mathematically, performance can be measured by the number of subsets used. Biologically, these numbers correspond to the amounts of resources used. While the Branch and Bound algorithm is capable of finding the optimal solution (albeit potentially over a long period), our observations show that the genetic algorithm can be as efficient as the Branch and Bound method with the correct choice of parameters. However, it may not be effective with some parameter choices, and the greedy algorithm generally performs worse.

Thus, we confirm our theoretical conclusion that sophisticated gene regulations in multicellular organisms can help to use resources more effectively than what is achievable in primitive cell colonies.

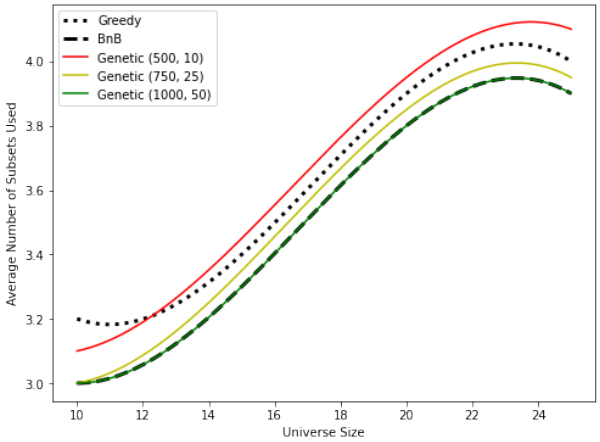

Figure A5: This figure compares the performance of different algorithms. Mathematically, their efficiency can be measured by the number of subsets used. Biologically, these numbers correspond to the amounts of resources utilized. Note that the Branch and Bound method finds the optimal solution, although the running time may be lengthy. We observe that the genetic algorithm can be as efficient as the Branch and Bound method with the correct choice of parameters; however, it can be ineffective with certain parameter choices, and the greedy algorithm performs worse in comparison.

References

[1] R. Donnell, ANALYSIS OF BOOLEAN FUNCTIONS, Cambridge University press, 2014.

[2] M. Gromov, A. Carbone, Mathematical slices of molecular biology, Preprint IHES/M/01/03 (2001) (2001).

[3] A. D. Ventsel, M. I. Freidlin, Random Perturbations of Dynamic Systems, Springer, New York, 1984.

[4] S. Vakulenko, D. Grigoriev, Deep gene networks and response to stress, Mathematics 9 (23) (2021) 3028. doi:https://doi.org/10.3390/math9233028.

[5] R. E. e. a. Lenski, Sustained fitness gains and variability in fitness trajectories in the long-term evolution experiment with escherichia coli, Royal Society Proceeding B B282 (2015) 20152292. doi:http://dx.doi.org/10.1098/rspb.2015.2292.

[6] K. Kishan, L. Rui, F. Cui, Q. Qi Yu, A. R. R. Haake, Gne: a deep learning framework for gene network inference by aggregating biological information,

BMC Systems Biology 13 (2019).
URL https://doi.org/10.1186/s12918-019-0694-y

[7] Z. Shen, H. Yang, S. Zhang, Nonlinear approximation via compositions (2019).
URL arXiv:1902.10170v4[cs.LG]

[8] W. E, Q. Wang, Exponential convergence of the deep neural network approximation for analytic functions, China Science Mathematics 61 (2018) 1733–1740.

[9] H. Montanelli, H. Yang, Q. Du, Deep relu networks overcome the curse of dimensionality for generalized bandlimited functions (2020).
URL arXiv:190300735v3[math.NA]

[10] J. J. Hopfield, Neural networks and physical systems with emergent collective computational abilities, Proc. Natl. Acad. Sci. 79 (8) (1982) 2554–2558. doi:10.1073/pnas.79.8.2554.
URL http://www.pnas.org/content/79/8/2554.shorthttp://www.pnas.org/cgi/doi/10.1073/pnas.79.8.2554

[11] C. Stern, Selection for subthreshold differences and the origin of pseudoexogenous adaptations, Am. Nat. 92 (866) (1958) 313–316.

[12] J. Reinitz, S. Vakulenko, D. Grigoriev, A. Weber, Adaptation, fitness landscape learning and fast evolution, F1000Research 8 (2019) 358. doi:10.12688/f1000research.18575.1.
URL https://f1000research.com/articles/8-358/v1

[13] P. Jiang, M. Kreitman, J. Reinitz, The relationship between robustness and evolution, bioRxiv (Feb. 2018). doi:10.1101/268862.
URL http://biorxiv.org/lookup/doi/10.1101/268862

[14] R. A. Watson, G. P. Wagner, M. Pavlicev, D. M. Weinreich, R. Mills, The evolution of phenotypic correlations and "developmental memory", Evolution 68 (4) (2014) 1124–1138. doi:10.1111/evo.12337.
URL http://doi.wiley.com/10.1111/evo.12337

[15] M. Talagrand, Spin glasses : a challenge for mathematicians : cavity and mean field models, Spinger, Berlin, New-York, 2003.

[16] M. Minsky, S. A. Papert, Perceptrons: An introduction to computational geometry, MIT press, 2017.

[17] A. R. Barron, Universal approximation bounds for superposition of a sigmoid function, IEEE Trans. Inf. Theory 39 (3) (1993) 930–945.

[18] C. Moore, S. Mertens, The Nature of Computation, Oxford University Press, 2011. doi:10.1093/acprof:oso/9780199233212.001.0001.
URL http://www.oxfordscholarship.com/view/10.1093/acprof:oso/9780199233212.001.0001/acprof-9780199233212

[19] J. Gravner, D. Pitman, S. Gavrilets, Percolation on fitness landscapes: Effects of correlation, phenotype, and incompatibilities, Journal of Theoretical Biology 248 (2007) 627–645. doi:10.1016/j.jtbi.2007.07.009.

[20] E. Friedgut, Sharp thresholds of graph properties, and the k-sat problem, J. Am. Math. Soc. 12 (04) (1999) 1017–1055. doi:10.1090/S0894-0347-99-00305-7.

[21] S. A. Cook, The complexity of theorem-proving procedures, in: Proc. third Annu. ACM Symp. Theory Comput. – STOC '71, ACM Press, New York, New York, USA, 1971, pp. 151–158. doi:10.1145/800157.805047.
URL http://dl.acm.org/citation.cfm?id=805047http://portal.acm.org/citation.cfm?doid=800157.805047

[22] L. A. Levin, Universal enumeration problems (russian), Probl. Peredai Inf. 9 (3) (1973) 115116.

[23] L. G. Valiant, Evolvability, Electron. Colloq. Comput. Complex. 120 (120) (2006) 1–19.
URL http://eccc.hpi-web.de/report/2006/120/

[24] E. Chastain, A. Livnat, C. Papadimitriou, U. Vazirani, Algorithms, games, and evolution, Proc. Natl. Acad. Sci. U. S. A. 111 (4) (2014) 1–4. doi:10.1073/pnas.1406556111.
URL http://www.ncbi.nlm.nih.gov/pubmed/24979793

[25] T. H. Cormen, C. E. Leiserson, R. L. Rivest, C. Stein, Introduction to Algorithms, MIT Press and McGraw-Hill, Cambridge, 2001.

[26] C. H. Papadimitriou, K. Steiglitz, Combinatorial optimization: algorithms and complexity, Dover. ISBN 0486402584, Mineola, NY, 1998.

[27] G. Sierksma, Y. Zwols, Linear and Integer Optimization: Theory and Practice, CRC Press. ISBN 978-1-498-71016-9, Boca Raton London New York, 2015.

[28] M. Mitchell, An Introduction to Genetic Algorithms, MIT Press. ISBN 9780585030944, Cambridge MA, 1996.

[29] M. Vose, The Simple Genetic Algorithm: Foundations and Theory, MIT Press. ISBN 978-0262220583, Cambridge MA, 1999.

[30] F. Jacob, J. Monod, Genetic regulatory mechanisms in the synthesis of proteins, Journal of Molecular Biology 3 (3) (1961) 318–356. doi:https://doi.org/10.1016/S0022-2836(61)80072-7.
URL https://www.sciencedirect.com/science/article/pii/S0022283661800727

[31] D. Achlioptas, A. Coja-Oghlan, Algorithmic barriers from phase transitions, in: Proceedings 49th FOCS, 2008, pp. 793–802.

[32] G. Caetano-Anollés, L. S. Yafremava, H. Gee, D. Derek Caetano-Anollés, H. S. Kim, J. E. Mittenthal, The origin and evolution of modern metabolism, Int J Biochem Cell Biol. 41 (2009) 285–97. doi:10.1016/j.biocel.2008.08.022.

[33] J. Maynard Smith, E. Schatzmary, The Major Transitions in Evolution, Oxford University Press, Oxford, 1995.

[34] S. Vakulenko, Complexity and Evolution of Dissipative Systems, De Gruyter Series in Mathematics and Life Sciences, Vol. 4, 2014.

[35] S. Vakulenko, D. Grigoriev, Stable growth of complex systems, in: Proceeding of Fifth Workshop on Simulation, 2005, pp. 705–709.

[36] R. Albert, A.-L. Barabási, Statistical mechanics of complex networks, Reviews of modern physics 74 (1) (2002) 47.
